# Supplementary material for: Phonon-driven wavefunction localization enhances room-temperature single-photon purity in large hybrid lead halide perovskite quantum dots
Source: Nat Commun. 2026 Jan 23;17:1974. doi: 10.1038/s41467-026-68607-w (PMC12932643; doi:10.1038/s41467-026-68607-w)
Supplement: Supplementary file 1 — Supplementary Information [file 41467_2026_68607_MOESM1_ESM.pdf]

# Phonon-driven wavefunction localization enhances room-temperature single-photon purity in large hybrid lead halide perovskite quantum dots

*Leon G. Feld,[1,2] Simon C. Boehme,[1,2] Sebastian Sabisch,[1,2] Nadav Frenkel,[3] Nuri Yazdani,[4] Viktoriia Morad,[1,2] Chenglian Zhu,[1,2] Taehee Kim,[1,2] Stefano Canossa,[1] Mariia Svyrydenko,[1,2] Rui Tao,[1,2] Maryna I. Bodnarchuk,[1,2] Gur Lubin,[3] Miri Kazes,[3] Vanessa Wood,[4] Dan Oron,[3,*] Gabriele Rainò[1,2,*] and Maksym V. Kovalenko[1,2,*]*

[1] Laboratory of Inorganic Chemistry, Department of Chemistry and Applied Biosciences, ETH Zürich, CH-8093 Zürich, Switzerland

[2] Laboratory for Thin Films and Photovoltaics, Empa – Swiss Federal Laboratories for Materials Science and Technology, CH-8600 Dübendorf, Switzerland

[3] Department of Molecular Chemistry and Materials Science, Weizmann Institute of Science, Rehovot 76100, Israel

[4] Department of Information Technology and Electrical Engineering, ETH Zürich, CH-8093 Zürich, Switzerland

Emails: mvkovalenko@ethz.ch, rainog@ethz.ch, dan.oron@weizmann.ac.il

Supplementary Information

Note 1. Quantum dot synthesis p. 2

Note 2. Photoluminescence quantum yield p. 5

Note 3. Single-crystal growth and X-ray scattering experiments p. 6

Note 4. Conventional single-particle optical spectroscopy p. 8

Note 5. Heralded single-particle optical spectroscopy p. 10

Note 6. Single-particle optical spectroscopy at 4 K p. 11

Note 7. Computational chemistry p. 12

Note 8. LUMO wavefunction localization p. 13

Supplementary Figures p. 14

**Supplementary Note 1: Quantum dot synthesis**

$FAPbBr_3$, $CsPbBr_3$, $MAPbBr_3$ and $FAPb(Br/Cl)_3$ QDs were synthesized following the methods described in ref.[1]. $FAPbI_3$ QDs were prepared according to ref.[2]. The synthesis of $AZPbBr_3$ QDs followed ref.[3]. Detailed descriptions of the syntheses are provided below.

1.1 $FAPbBr_3$ QDs

*$PbBr_2$-TOPO precursor, 0.067M*. Lead(II) bromide (0.2 mmol, 99.998%, Alfa) and trioctylphosphine oxide (1 mmol, 90%, STREM), were dissolved in 2.5 ml n-octane (>99% for synthesis, Roth) at 120°C on a hotplate in air.

*$PbBr_2$-TOPO precursor, 0.04M*. Lead(II) bromide (1 mmol, 99.999% Sigma-Aldrich) and trioctylphosphine oxide (5 mmol, 90%, STREM), were dissolved in 5 ml n-octane (>99% for synthesis, Roth) at 120°C on a hotplate in air, cooled to room temperature, filtered through a syringe filter and dilute in 20 ml of n-hexane (>97%, HPLC, Sigma-Aldrich).

*FA-DOPA-OA precursor, 0.06M*. Formamidine acetate (0.6 mmol, 99%, abcr), diisooctylphosphinic acid (3ml, 90%, Sigma-Aldrich) and oleic acid (2 ml, 90% Sigma-Aldrich) added in 5 ml n-octane (>99% for synthesis, Roth) and heated to 120°C in air until the salt dissolves.

*2-octyldodecylphosphoethanolamine* (C8C12-PEA) ligand has been synthesized according to ref.[1]

*~6 nm $FAPbBr_3$ QDs*. $PbBr_2$-TOPO precursor (520 µL, 0.067M) diluted with n-hexane (62.5 ml) stirred in an open flat-bottom flask on a stirring plate. To this solution, FA-DOPA-OA precursor (200 µL, 0.06M) was swiftly injected, followed by immediate addition of C8C12-PEA ligand (5 mg in 50 µL of mesitylene). Resulting QDs are purified by addition of 3 eq. of antisolvent (ethylacetate:acetonitrile, 2:1 v:v), centrifugation (at maximum centrifugation speed for 30s) and redispersion of the precipitate in n-hexane. The purification was repeated two times in total.

*~8.5 nm $FAPbBr_3$ QDs*. $PbBr_2$-TOPO precursor (260 µL, 0.067M) diluted with n-hexane (1.5 ml) was stirred in an open vial on a stirring plate. To this solution, FA-DOPA-OA precursor (100 µL, 0.06M) was swiftly injected, followed by immediate addition of C8C12-PEA ligand (5 mg in 50 µL of mesitylene). Resulting QDs were purified by addition of 2 eq. of antisolvent (ethylacetate:acetonitrile, 2:1 v:v), centrifugation (at maximum centrifugation speed for 30s) and redispersion of the precipitate in n-hexane. The purification was repeated two times in total.

*~10 nm $FAPbBr_3$ QDs*. $PbBr_2$-TOPO precursor (2 ml, 0.04M) diluted with n-hexane (12 ml) stirred in a round bottom flask on a stirring plate. To this solution, FA-DOPA-OA precursor (0.4 ml, 0.06M) is swiftly injected, followed 15 seconds later by addition of C8C12-PEA ligand (20 mg in 200 µL chloroform/methanol 9:1 v:v). Resulting QDs are purified by addition of 3 eq. of antisolvent (ethylacetate:acetonitrile, 2:1 v:v), centrifugation at 12.1 krpm (20130 × g) for 1 min and redispersion of the precipitate in n-hexane. The purification was repeated three times in total.

*~17 nm $FAPbBr_3$ QDs*. FA-DOPA-OA and $PbBr_2$-TOPO precursor solutions were slowly injected, followed by the addition of C8C12-PEA ligand. Resulting QDs were purified by addition of 3 eq. of antisolvent (ethylacetate:acetonitrile, 2:1 v:v), centrifugation at 12.1 krpm (20130 × g) for 1 min and redispersion of the precipitate in cyclohexane. The purification was repeated once.

1.2 $CsPbBr_3$ QDs

*$PbBr_2$-TOPO precursor, 0.067M*. Lead(II) bromide (0.2 mmol, 99.998%, Alfa) and trioctylphosphine oxide (1 mmol, 90%, STREM), were dissolved in 2.5 ml n-octane (>99% for synthesis, Roth) at 120°C on a hotplate in air.

*$PbBr_2$-TOPO precursor, 0.04M*. Lead(II) bromide (1 mmol, 99.999% Sigma-Aldrich) and trioctylphosphine oxide (5 mmol, 90%, STREM), were dissolved in 5 ml n-octane (>99% for synthesis, Roth) at 120°C on a hotplate in air, cooled to room temperature, filtered through a syringe filter and dilute in 20 ml of n-hexane (>97%, HPLC, Sigma-Aldrich).

*Cs-DOPA precursor, 0.02M*. Cesium carbonate (0.3 mmol, 99.9% trace metals basis, Sigma-Aldrich) and diisooctylphosphinic acid (1 ml) were mixed in n-octane (2 ml, >99% for synthesis, Roth) and heated to 120°C in air until the salt dissolves and gas evolution stops. After cooling to room temperature, the solution is diluted with n-hexane (27 ml, >97%, HPLC, Sigma-Aldrich) to reach 0.02M concentration.

*Cs-BTPPA precursor, 0.02M*. Cesium carbonate (0.3 mmol, 99.9% trace metals basis, Sigma-Aldrich) and bis(2,4,4-trimethylpentyl)phosphinic acid (1 ml, 90%, Fluorochem) were mixed in n-octane (2 ml, >99% for synthesis, Roth) and heated to 120°C in air until the salt dissolves and gas evolution stops. After cooling to room temperature, the solution is diluted with n-hexane (27 ml, >97%, HPLC, Sigma-Aldrich) to reach 0.02M concentration and filtered through a syringe filter.

*2-octyldodecylphosphoethanolamine* (C8C12-PEA) ligand has been synthesized according to ref.[1]

*~7.5 nm $CsPbBr_3$ QDs*. $PbBr_2$-TOPO precursor (2 ml, 0.04M) was diluted in 40 ml vial with 6 ml of n-hexane and Cs-BTPPA precursor (2 ml, 0.02M) was injected under vigorous stirring at room temperature. After 10 minutes, C8C12-PEA ligand (100 µL, 0.1mg/µL in mesitylene) was added. QDs were purified by addition of 3 eq. of antisolvent (acetone), centrifugation at 12.1 krpm (20130 × g) for 1 min, and redispersion in hexane. The purification was repeated three times in total, and the final precipitate was redispersed in cyclohexane.

*~10 nm $CsPbBr_3$ QDs.* $PbBr_2$-TOPO precursor (4 ml, 0.04M) were added to 25 ml round bottom flask and Cs-DOPA precursor (2 ml, 0.02M) was injected under vigorous stirring at room temperature. After 150 seconds, C8C12-PEA ligand (75 µL, 0.1mg/µL in mesitylene) was added. QDs were purified by addition of 3 eq. of antisolvent (ethylacetate:acetonitrile, 2:1 v:v), centrifugation at 12.1 krpm (20130 × g) for 1 min and redispersion in cyclohexane.

*~17 nm $CsPbBr_3$ QDs*. Cs-OA and $PbBr_2$-TOPO precursor solutions were slowly injected, followed by the addition of C8C12-PEA ligand. Resulting QDs were purified by addition of 3 eq. of antisolvent (ethylacetate:acetonitrile, 2:1 v:v), centrifugation at 12.1 krpm (20130 × g) for 1 min and redispersion of the precipitate in cyclohexane. The purification was repeated once.

1.3 $FAPb(Br/Cl)_3$ QDs

*$PbBr_2$-TOPO precursor, 0.067M*. Lead(II) bromide (0.2 mmol, 99.998%, Alfa) and trioctylphosphine oxide (1 mmol, 90%, STREM), were dissolved in 2.5 ml n-octane (>99% for synthesis, Roth) at 120°C on a hotplate in air.

$ZnCl_2$-TOPO precursor, 0.067M. Zinc chloride (0.2 mmol, puriss., anhydrous, ≥ 98 %, Sigma-Aldrich) and trioctylphosphine oxide (1 mmol, 90%, STREM), were dissolved in n-octane (2.5 ml, >99% for synthesis, Roth) at 120°C on a hotplate in air un.

*FA-DOPA-OA precursor, 0.06M*. Formamidine acetate (0.6 mmol, 99%, abcr), diisooctylphosphinic acid (3ml, 90%, Sigma-Aldrich) and oleic acid (2 ml, 90% Sigma-Aldrich) added in 5 ml n-octane (>99% for synthesis, Roth) and heated to 120°C in air until the salt dissolves.

*2-octyldodecylphosphoethanolamine* (C8C12-PEA) ligand has been synthesized according to ref.[1]

*$FAPb(Br/Cl)_3$ QDs.* $PbBr_2$-TOPO precursor (260 µL, 0.067M) mixed with $ZnCl_2$-TOPO precursor in n-hexane (5 ml) in an open vial on a stirring plate. To this solution, FA-DOPA-OA precursor (100 µL, 0.06M) is swiftly injected. After 5 seconds, C8C12-PEA ligand (5 mg in 100 µL of mesitylene) is added. QDs are purified by addition of 2 eq. of antisolvent (ethylacetate:acetonitrile, 2:1 v:v), centrifugation (at maximum centrifugation speed for 30s) and redispersion of the precipitate in n-hexane. The purification was repeated two times in total.

1.4 $FAPbI_3$ QDs

*Preparation of formamidinium oleate*. Formamidine acetate (2.5 mmol, 0.26 g, Aldrich, 99%) was loaded into a 50 mL three-neck flask along with hexadecane (12 mL) and oleic acid (8 mL, Sigma-Aldrich, 90%). The reaction mixture was degassed three times at room temperature, heated to 100 °C under nitrogen until the reaction was completed, and then cooled to room temperature. The solution was stored in a glovebox.

*Synthesis of $FAPbI_3$ QDs.* In a 25 mL three-necked flask was suspended lead(II) iodide (61 mg, 0.133 mmol, Alfa Aesar 99,9998 %) in hexadecane (4.6 mL), and the mixture was heated to 60 °C and dried under vacuum for 20 min. Then the reaction mixture was heated to 110 °C under nitrogen, and distilled oleylamine (0.5 mL, STREM) and dried oleic acid (1.0 mL, Sigma-Aldrich, 90%) were injected. Once the lead(II) iodide dissolved, the reaction mixture was cooled to 80 °C, the mixture of formamidinium oleate (5.0 mL) and dried hexadecane (1.0 mL) was injected, and 15 s later, the reaction mixture was cooled by a water bath.

*Purification of $FAPbI_3$ QDs*. The crude solution was centrifuged at 12.1 krpm (20130 × g) for 5 min, and the supernatant was discarded. The precipitate was dissolved in anhydrous hexane (0.3 mL), and the solution was centrifuged again at 12.1 krpm (20130 × g) for 5 min. Precipitate was discarded and 0.2 mL of anhydrous hexane was added to the supernatant. To the hexane solution of QDs were added anhydrous hexane (0.5 mL), anhydrous toluene (1 mL), and anhydrous methyl acetate (2.15 mL). The solution was centrifuged at 13.4 krpm (12100 × g) for 3 min, and the precipitate was dissolved in 1 mL anhydrous toluene following filtration through a 0.45 mm PTFE filter.

1.5 $AZPbBr_3$ QDs

*Aziridine stock solution* (0.15 M) was prepared as follows: 15.5 µL of aziridine (Ochem Incorporation) was dissolved in 2 mL of anhydrous chloroform and stored in a refrigerator. For *$PbBr_2$-TOPO stock solution* (0.04 M), PbBr2 (1 mmol, 376 mg, 99.999%, Sigma-Aldrich) and trioctylphosphine oxide (TOPO, Strem 90%, 5 mmol, 2.15 g) were dissolved in octane (5 mL) at 100 °C, followed by dilution with hexane (20 mL) and filtering through a 0.2 µL PTFE filter before use. *Diisooctylphosphinic acid (DOPA) stock solution* (0.57 M) was prepared by dissolving 0.8 mL of DOPA (Sigma-Aldrich) in 3.2 mL hexane.

*Oleic acid (OAc) stock solution* (0.515 M) was prepared by dissolving 0.8 mL of OAc (Sigma-Aldrich, 90%) in 3.6 mL of hexane. *2-octyldodecylphosphoethanolamine stock solution* (50 mg/mL) was prepared by dissolving 100 mg of 2-octyldodecylphosphoethanolamine in 2 mL of distilled mesitylene. 2-octyldodecylphosphoethanolamine has been synthesized according to ref.[1].

*Synthesis of ~10 nm AZPbBr$_3$ QDs.* In a 25 mL one-neck flask, 0.65 mL PbBr2-TOPO stock solution was combined with 3 mL hexane. Then 0.1 mL of DOPA and 0.2 mL OAc stock solutions were added. Under vigorous stirring (1100 rpm), 44 µL aziridine in chloroform was swiftly injected into the reaction mixture. After 30 s, 15 µL of 2-octyldodecylphosphoethanolamine ligands is added to initiate the ligand exchange on the NC surface. In 2 min after the addition of the ligands, the crude solution was concentrated by evaporating hexane on a rotary evaporator down to <0.5 mL of residual solvent. The QDs were precipitated from the concentrated colloid by adding acetonitrile (crude solution: nonsolvent 1:1 (v/v)), centrifuged at 13.4 krpm (12100 × g) for 2 min and redissolved in 0.5 mL anhydrous toluene.

1.6 MAPbBr$_3$ QDs

*PbBr$_2$-TOPO precursor, 0.067M*. Lead(II) bromide (0.2 mmol, 99.998%, Alfa) and trioctylphosphine oxide (1 mmol, 90%, STREM), were dissolved in 2.5 ml n-octane (>99% for synthesis, Roth) at 120°C on a hotplate in air.

*MA-DOPA-OA precursor, 0.06M*. Methylamine (90 µL; 1M in THF, Flurochem) mixed with diisooctylphosphinic acid (900 µL, 90%, Sigmal-Aldrich) and oleic acid (600 µL, 90%, Sigmal-Aldrich) in n-octane (1.5 ml, >99% for synthesis, Roth) at room temperature on a stirring plate.

*2-octyldodecylphosphoethanolamine* (C8C12-PEA) ligand has been synthesized according to ref.[1]

*~9 nm MAPbBr$_3$ QDs*. PbBr$_2$-TOPO precursor (520 µL, 0.067M) diluted in n-hexane (6 ml) in an open vial on a stirring plate. To this solution, MA-DOPA-OA precursor (200 µL, 0.06M) is swiftly injected. Immediately, C8C12-PEA ligand (5 mg in 100 µL of mesitylene) is added. QDs are purified by addition of 3 eq. of antisolvent (ethylacetate:acetonitrile, 2:1 v:v), centrifugation (at maximum centrifugation speed for 30s) and redispersion of the precipitate in n-hexane. The purification was repeated three times in total.

**Supplementary Note 2: Photoluminescence quantum yield**

The photoluminescence quantum yield of the solutions was measured with a quantum yield spectrometer equipped with an integrating sphere (Hamamatsu, Model Quantaurus-QY Absolute PL).

**Supplementary Note 3: Single-crystal growth and X-ray scattering experiments**

3.1 $FAPbBr_3$ crystal growth

Single crystals of $FAPbBr_3$ were grown from a 1.4 M solution of FABr (Greatcell Solar, >99.99%) and $PbBr_2$ (99.999% Sigma-Aldrich) in a 1:1 volumetric mixture of dimethylformamide (Sigma-Aldrich, >99%) and gamma-butyrolactone (Arcos Organics, >99%). The solution was filtered through a 0.22 µm PTFE syringe filter into a 4 mL glass vial. The vial was placed in an oil bath which was slowly heated from room temperature to 60 °C under ambient conditions inducing crystallization. After the formation of the initial crystallites, the solution was heated to 70 °C for 4 hours. The resulting cm-sized crystals were extracted, patted dry and washed three times in toluene (Sigma-Aldrich, >99.7%) to remove remaining starting materials. The resulting crystals were optically transparent and showed a dark-red colour.

3.2 $CsPbBr_3$ crystal growth

Single crystals of $CsPbBr_3$ were grown from a 1M solution of CsBr (Chemcraft, >99.999%) and $PbBr_2$ (Sigma-Aldrich, 99.999%) in DMSO (Sigma-Aldrich, >99.5%). After full dissolution at 50 °C, the solution was filtered through a 0.22 µm PTFE syringe filter into a 4 mL glass vial. The vial was then heated to 100 °C to induce crystallization. The temperature was kept constant until the desired crystal size was reached. The crystals were then extracted from the solution, dried and washed with toluene three times (Sigma-Aldrich, >99.7%). The resulting crystals showed a bright orange colour.

3.3 Single-crystal X-ray diffraction

Structure solution and refinement were conducted using the software Olex2[4]. Solution was achieved by the program ShelXT[5] included in the Olex2 software suite (Intrinsic phasing method). Refinement was based on the Program ShelXL[6], using the least-square algorithm.

Every atom has been unambiguously identified and modelled by allowing for anisotropic displacement parameters refinement. In the case of $CsPbBr_3$, a minor merohedral twinning was found with twin law (0.0, 0.0, 1.0, 0.0, -1.0, 0.0, 1.0, 0.0, 0.0), corresponding to ca. 8% of the intensity contribution. More information can be found in the deposited cif files (Supporting Data) and Tables S1-S3. Structures including structure factors information can be accessed in the CSD and ICSD databases by referring to the deposition numbers 2407691 ($FAPbBr_3$) and 2407795 ($CsPbBr_3$). Crystal structures were visualized using VESTA[7].

Supplementary Table 1: Full details of the single-crystal XRD structure solutions of $FAPbBr_3$ and $CsPbBr_3$.

| Sample / Empirical Formula | **$FAPbBr_3$** | **$CsPbBr_3$** |
|---|---|---|
| Formula weight | 491.99 | 579.83 |
| Temperature | 300.0(3) K | 295.15 K |
| Crystal system | cubic | orthorhombic |
| Space group | *Pm*-3*m* | *Pnma* |
| a | 5.99900(10) Å | 8.2575(4) Å |
| b | a | 11.7639(7) Å |

| | | |
|---|---|---|
| c | a | 8.2194(4) Å |
| α = β = γ | 90 ° | 90 ° |
| Volume | 215.892(11) $Å^3$ | 798.44(7) $Å^3$ |
| ρ | 3.784 $g/cm^3$ | 4.824 $g/cm^3$ |
| μ | 33.329 $mm^{-1}$ | 40.519 $mm^{-1}$ |
| F(000) | 212.0 | 968.0 |
| Crystal size | 0.07 × 0.07 × 0.05 $mm^3$ | 0.064 × 0.053 × 0.033 $mm^3$ |
| Radiation | Mo Kα (λ = 0.71073 Å) | Mo Kα (λ = 0.71073 Å) |
| 2Θ range | 6.792° to 58.952° | 6.028° to 60.946° |
| Index ranges | -8 ≤ h ≤ 8, -8 ≤ k ≤ 8, -8 ≤ l ≤ 7 | -11 ≤ h ≤ 11, -15 ≤ k ≤ 15, -10 ≤ l ≤ 10 |
| Reflections collected | 1681 | 12744 |
| $R_{int}$ | 0.0196 | 0.0373 |
| $R_{sigma}$ | 0.0056 | 0.0163 |
| Data/restraints/parameters | 88/11/10 | 1178/18/42 |
| GoF on $F^2$ | 1.181 | 1.077 |
| R indices (I > 2σ(I)) | $R_1$ = 0.0089, $wR_2$ = 0.0228 | $R_1$ = 0.0260, $wR_2$ = 0.0675 |
| R indices (all data) | $R_1$ = 0.0089, $wR_2$ = 0.0228 | $R_1$ = 0.0306, $wR_2$ = 0.0698 |
| Largest diff. peak/hole | 0.24/-0.24 $eÅ^{-3}$ | 1.44/-1.26 $eÅ^{-3}$ |

Supplementary Table 2: Fractional atomic coordinates ($x10^4$) and equivalent isotropic displacement parameters in $Å^2x10^3$.

| | x | y | z | U(eq) |
|---|---|---|---|---|
| **$FAPbBr_3$** | | | | |
| Pb | 5000 | 5000 | 5000 | 40.80(15) |
| Br | 0 | 5000 | 5000 | 86.9(3) |
| C | 0 | 0 | 0 | 145(11) |
| N | 2050.09 | -711.39 | 0 | 120(20) |
| **$CsPbBr_3$** | | | | |
| Pb01 | 0 | 5000 | 5000 | 28.47(12) |
| Cs02 | 5537(16) | 2500 | 4806(17) | 55.4(13) |
| Br03 | 2933.1(11) | 4756.2(9) | 2938.8(11) | 69.2(3) |
| Cs | 5240(30) | 2500 | 4850(20) | 74(3) |
| Br1 | -37.4(18) | 2500 | 5472(2) | 79.0(5) |
| Cs1 | 5070(30) | 2500 | 5184(18) | 77(3) |

Supplementary Table 3: Anisotropic displacement parameters in $Å^2x10^3$.

| | $U_{11}$ | $U_{22}$ | $U_{33}$ | $U_{23}$ | $U_{13}$ | $U_{12}$ |
|---|---|---|---|---|---|---|
| **$CN_2H_5PbBr_3$** | | | | | | |
| Pb | 40.80(15) | 40.80(15) | 40.80(15) | 0 | 0 | 0 |
| Br | 36.5(3) | 112.1(4) | 112.1(4) | 0 | 0 | 0 |
| C | 145(11) | 145(11) | 145(11) | 0 | 0 | 0 |

| | | | | | | |
|---|---|---|---|---|---|---|
| N | 88(13) | 140(50) | 120(30) | 0 | 0 | -9(19) |
| **$CsPbBr_3$** | | | | | | |
| Pb01 | 29.38(19) | 26.89(17) | 29.1(2) | -0.74(8) | 0.01(10) | 0.84(8) |
| Cs02 | 54(3) | 43.0(18) | 69(3) | 0 | -11(2) | 0 |
| Br03 | 56.4(5) | 94.2(6) | 56.9(5) | 5.9(4) | 30.2(4) | 5.8(5) |
| Cs | 69(6) | 85(4) | 66(5) | 0 | -1(4) | 0 |
| Br1 | 113.6(13) | 24.5(5) | 98.8(10) | 0 | -11.4(7) | 0 |
| Cs1 | 79(6) | 79(3) | 72(6) | 0 | -19(4) | 0 |

3.4 Single-crystal total scattering measurements

For total scattering measurements, selected specimens were mounted on a MiTeGen kapton loop using inert NVH high viscosity oil. Diffraction data were collected by a Rigaku Synergy S diffractometer equipped with a Pilatus 300K hybrid pixel detector, Mo Kalpha microfocus source, and an Oxford Cryostream system set at a temperature of 300K. Images were acquired in a 180 degrees rotation scan with rotation axis orthogonal to the primary beam, and detector in 2 Theta position = 0. A fine slicing of reciprocal space was achieved by adopting a rotation step between subsequent frames of 0.2 degrees.

The resulting data was processed by the program XDS[8] to determine the crystal orientation matrix, and the program Meerkat (https://github.com/aglie/meerkat) to produce the 3D reciprocal space reconstructions. Both phases were indexed by using the unit cell metric of $FAPbBr_3$ (refined in each separate case by XDS) to allow for a meaningful comparison between reconstructions. These were based on h,k,l maximum index of 10, and a reciprocal space step of 0.05 reciprocal lattice units, resulting in volumes of 401x401x401 voxels. Final corrections such as symmetry averaging (Laue symmetry m-3m for $FAPbBr_3$, mmm for $CsPbBr_3$) and isotropic background subtraction were conducted with a custom python-based script. Reciprocal space reconstructions for both $FAPbBr_3$ and $CsPbBr_3$ have been normalized by dividing each voxel's value by the total number of counts in the reconstruction, then multiplying the obtained values by a constant ($10^9$). For data visualization and extraction of intensity profiles the program Nexpy was used (https://nexpy.github.io/nexpy/).

**Supplementary Note 4: Conventional single-particle spectroscopy**

4.1 Sample preparation

Sample preparations were performed in nitrogen filled gloveboxes using anhydrous and filtered solvents. As-synthesized $FAPbBr_3$, $CsPbBr_3$ and $FAPb(Br/Cl)_3$ QDs samples were diluted in multiple steps by three to five orders of magnitude in cyclohexane (ACROS, 99.5% extra dry over mol. sieves) or n-octane (ACROS, 99+% extra dry) and spin-coated (100 µL, 150 revolutions per second, 60 s) onto clean precision cover glasses (Thorlabs, 170 ± 5 µm thickness, Ø25 mm diameter). Additionally, prior to spin-coating, most samples were additionally diluted by one to two orders of magnitude in 3 mass% solution of Polystyrol-*block*-poly(ethylen-*ran*-butylen)-*block*-polystyrol (SEBS; ALDRICH, average $M_w \approx 1.18 \times 10^5$) in cyclohexane for additional photostability. As-synthesized $FAPbI_3$ QDs were diluted in multiple steps by three orders of magnitude in toluene (ACROS, 99.85% extra dry over mol. Sieves)

before further diluting by one order of magnitude in a solution of 3 mass% of polystyrene (ALDRICH, average $M_w \approx 2.8 \times 10^5$) and spin-coating.

The resulting sparse QD films were placed in a nitrogen filled sample holder.

4.2 Data processing

*Photoluminescence spectra* recorded by the EMCCD camera were imported using the spe2py package (https://github.com/ashirsch/spe2py) and analysed with custom-built python codes. To determine the PL energy $E_{max}$, the first 5 frames of the PL spectra series were integrated and smoothed (gaussian filter, 0.3 nm width) and the wavelength at the PL maximum was determined. The slope energy $E_{slope}$ was obtained by fitting the red tail of the spectrum $I(E, E < E_{max})$:

$$I(E, E < E_{max}) = I_0 e^{\frac{E-E_{max}}{E_{slope}}} \qquad \text{(eq. S1)}$$

*Time-tagged data* generated in the Hanbury-Brown and Twiss experiment was mostly analysed in python utilizing the pycorrelate package (https://github.com/OpenSMFS/pycorrelate) to compute second-order correlation functions $g^2(\tau)$. In some cases, second-order correlation functions were constructed online in start-stop operation. For the power-dependent $g^2(\tau)$, the SymPhoTime 64 software (PicoQuant) was used to facilitate the selection of a specific time-window corresponding to a fixed power range. The $g^2(\tau)$ was fitted with biexponential decays to account for the fast and slowly decaying components that are observed in perovskite QDs:

$$g^2(\tau) = \sum_{k=-n}^{n} A_k \left( \frac{1}{1+r} e^{-\left|\frac{\tau-\left(\frac{k}{f_{exc}}\right)}{\tau_A}\right|} + \left(1 - \frac{1}{1+r}\right) e^{-\left|\frac{\tau-\left(\frac{k}{f_{exc}}\right)}{\tau_B}\right|} \right) \qquad \text{(eq. S2)}$$

Here, $n$ is the number of pulses fitted on both sides of the peak at $\tau = 0$ (usually $n$ is 2 or 3), $A_k$ are the amplitudes of the peaks, $f_{exc}$ is the pulse repetition rate of the excitation and $r$ defines the fraction of photons decaying with the lifetimes $\tau_A$ and $\tau_B$ respectively. The anti-bunching $g^2(0)$ is then determined from the ratio of $A_0$ and the mean value of all other $A_k$.

*The emission saturation curves* were obtained by recording photon-arrival times on the APDs while changing the excitation power with a neutral-density filter on a linear motorized stage. The power was first increased and then decreased to ensure that QDs did not photobleach during irradiation. The photon arrival times were binned (1 ms bin width) and classified in to ON and OFF events. A bin was considered an OFF event if its counts were smaller than half the average counts within 2 seconds. The bins corresponding the ON events were fitted with a model accounting for exciton and biexciton emission:[9]

$$I(P) = A\left(1 - e^{-\frac{P}{P_{sat}}}\right) + B\,\frac{P}{P_{sat}} \qquad \text{(eq. S3)}$$

*Blinking traces* were obtained by binning the photon-arrival times recorded on both APDs with a bin width of 1 ms.

**Supplementary Note 5: Heralded single-particle spectroscopy**

Cascaded emission events are directly probed at room temperature, extracting both temporal and spectral information simultaneously. Using heralded spectroscopy, single particles are excited by a pulsed laser, dispersing the emitted fluorescence by a grating spectrometer, and detecting the photons (temporally and spectrally resolved) with a single-photon avalanche diode (SPAD) array detector. Only photon-pair emissions detected following the same excitation pulse are post-selected and treated as heralded events. Each photon within the post-selected photon pairs is time- and energy-tagged according to its time and pixel of detection. The high spectral and temporal resolutions enable an unambiguous temporal separation between the two detections, attributing the first arriving photon to emission from the biexciton (BX) state and the second photon to emission from the exciton (1X) state.

5.1 Sample preparation

In a nitrogen-filled glovebox, QDs dispersed in cyclohexane were diluted by a factor of 3000 in cyclohexane (ALDRICH, 99.5% anhydrous) followed by further dilution by a factor of 100 in a 3 mass% solution of Polystyrol-*block*-poly(ethylen-*ran*-butylen)-*block*-polystyrol (SEBS; ALDRICH, average $M_w \approx 1.18 \times 10^5$) in cyclohexane. 100 µL of the resulting solution were spin-coated onto clean cover glasses at 150 revolutions per second for 60 s. Final films were measured under ambient conditions.

5.2 Setup

The SPAD array spectrometer previously described in refs.[10,11] is built around a commercial inverted microscope (Eclipse Ti-U, Nikon). Excitation light from a pulsed laser source (470 nm, 5 MHz, LDH-P-C-470B, PicoQuant, $\langle N \rangle < 0.08$) is focused onto the sample with an oil immersion objective (×100, 1.3 NA, Nikon). The emitted light is collected by the same objective and passed through a dichroic mirror (FF484-FDi02-t3, Semrock) and a long-pass filter (BLP01-473R, Semrock). The magnified image plane (×150) serves as the input for a Czerny-Turner spectrometer that consists of a 4-f system (AC254-300-A-ML and AC254-100-A-ML, Thorlabs) with a blazed grating (53-*-201R, Richardson) at the Fourier plane. At the output image plane of the spectrometer, a 512-pixel on-chip linear SPAD array is placed. Only fixed quarters of 64 pixels can participate simultaneously in the time-tagging measurement, which is done by an array of 64 time-to-digital converters (TDCs) implemented on a field programmable gate array (FPGA). The physical pixel pitch is 26.2 um, which corresponds to a difference between neighboring pixels of ~1.7 nm in wavelength. Of the single 64-pixel segment used in this work, the 34th pixel is a `hot' pixel and therefore omitted from all analyses. The instrument response function (IRF) of the system featured a ~190 ps full width at half maximum (FWHM). The pixels' dead time is ~15 ns and the average dark counts are ~41 counts per second (CPS) per pixel.

5.3 Data processing

*Second-order correlation function.* The $g^2(0)$ was calculated and corrected for errors emanating from dark counts and inter-pixel crosstalk according to the protocol detailed in ref.[12].

*Identification of photon pairs.* Sequential photon emissions that were both detected after the same laser excitation pulse were registered as heralded events, providing that they met the following temporal and pixel constraints. The first detected photon of the pair (BX) was constrained between -0.5 and 10 ns delay from the laser pulse peak. The lower gate is to avoid the exclusion of as many events as possible. The second photon of the pair (1X) was gated between 0.5 to 60 ns delay from the first photon. The non-zero lower gate here was to diminish the number of false events caused by inter-

pixel crosstalk. The upper bounds of both photons are longer than their respective lifetimes but significantly shorter than the laser pulse period (200 ns). This was chosen to lower signal loss while maintaining low dark counts contributions and ensuring both photons originated from the same excitation pulse. In addition, because of the detection dead time sequential detections in the same pixel could only occur if the photons are 15 ns apart or longer. Therefore, photon pairs that were detected at the same pixel were excluded entirely to prevent bias in favour of longer-lived photon cascades. After sifting the raw data for cascaded BX-1X events, statistical corrections for dark counts and crosstalk were applied to subtract false detections.[10,11]

*Lifetime Assessment.* The BX and 1X detections are binned to 0.25 ns bins according to their delay from the laser pulse to form a 1D temporal decay histogram for each (BX and 1X). The decay lifetimes are then assessed as the temporal delay from the laser pulse where the population drops by 1/e.

*Spectral Peak Assessment.* A 1D spectral histogram of both the BX and 1X detections is formed by binning them to the 64 SPAD pixels, indicating the photon energies. The spectrum of each (BX and 1X) is then fitted to a Cauchy–Lorentz distribution, where the distribution peaks ($E_{1X} \backslash E_{BX}$) serve in the calculation of the BX shift; $\Delta_{BX} \equiv E_{1X} - E_{BX}$.

**Supplementary Note 6: Single-particle spectroscopy at 4 K**

6.1 Sample preparation

The sample preparation followed the description in section 5.1, but the solutions were spin-coated onto a $SiO_2$/Si substrate and placed in the cryostat, followed by evacuation and cool-down.

6.2 Experiments

After optimizing the stage position for the maximum signal from single QD, PL spectra were recorded with 1 s integration time, with and without the tunable band-pass filter (TBP01-547/15, Semrock) to filter out the biexciton emission. Then, the single QD PL was sent to the SPDs to record the second-order intensity correlation ($g^2$), again with and without filtering the biexciton emission. Excitation power-dependent PL spectra were obtained by recording the PL spectra with 2 s integration time, moving the linear-motorized neutral density filter to sweep the laser power from <30 nJ/cm$^2$ to ~6 uJ/cm$^2$.

6.3 Data processing

*Photoluminescence spectra* were plotted by averaging over 5-10 frames and normalizing to the peak maximum.

*Power dependence* of each emission line was obtained by fitting each frame of the PL spectra (2s integration) with Lorentzian functions and extracting the corresponding peak areas.

*Time-tagged data* generated in the Hanbury-Brown and Twiss experiment was analysed in python utilizing the pycorrelate package (https://github.com/OpenSMFS/pycorrelate) to compute second-order correlation functions $g^2(\tau)$. The $g^2(\tau)$ was fitted with monoexponential decays with a constant offset describing the fast decay observed in perovskite QDs at low temperature as well as the quasi-continuous background observed in our experiments:

$$g^2(\tau) = \text{bg} + \sum_{k=-n}^{n} A_k e^{-\left|\frac{\tau - \left(\frac{k}{f_{exc}}\right)}{\tau_A}\right|} \qquad \text{(eq. S4)}$$

Here, $n$ is the number of pulses fitted on both sides of the peak at $\tau = 0$ (usually $n$ is 7), $A_k$ are the amplitudes of the peaks, $f_{exc}$ is the pulse repetition rate of the excitation and bg is the constant offset. The anti-bunching $g^2(0)$ is then determined from the ratio of $A_0$ and the mean value of all other $A_k$.

**Supplementary Note 7: Computational chemistry**

7.1 Isosurfaces of wavefunctions

Images of wavefunction isosurfaces were obtained from cube files using VESTA (https://jp-minerals.org/vesta).

7.2 Wavefunction size

To determine the wavefunction size from the raster image in the cube files, we first compute the variance of the wavefunction density $D_{i,j,k}$, where i, j and k run over the pixels, along the x, y and z direction:

$$\sigma_x^2 = \frac{1}{N_x} \sum_{i=1}^{N_x} (x_i - \bar{x})^2 \sum_{j,k} D_{i,j,k} \qquad \text{(eq. S5)}$$

Here, $N_x$ corresponds to the number of pixels along the x-direction and $\bar{x}$ is the center of the wavefunction

$$\bar{x} = \frac{1}{N_x} \sum_{i=1}^{N_x} x_i \sum_{j,k} D_{i,j,k} \ . \qquad \text{(eq. S6)}$$

The wavefunction size is then defined as the full width at half maximum (FWHM):

$$\text{FWHM} = 2.355 \times \sqrt{\frac{\sigma_x^2 + \sigma_y^2 + \sigma_z^2}{3}} \qquad \text{(eq. S7)}$$

7.3 Wavefunction autocorrelation and power spectrum

The autocorrelation function ($C_\Phi(\tau)$) of the wavefunction $\mathbf{\Phi}(t)$ in an orthogonal basis can be rewritten as

$$C_\Phi(\tau) = \langle \mathbf{\Phi}(t+\tau) | \mathbf{\Phi}(t) \rangle_t = \int_{-\infty}^{\infty} dt \sum_i c_i(t) c_i^*(t+\tau) \quad \text{(eq. S8)}$$

Here, $c_i(t)$ are the MO coefficients at time $t$. Due to the arbitrary sign-switching of the coefficients in DFT, $c_i(t)$ are replaced by their absolute value and $C_\Phi(\tau)$ becomes

$$C_\Phi(\tau) = \int_{-\infty}^{\infty} dt \sum_i (|c_i(t)| - \langle c_i \rangle_t)\,(|c_i(t+\tau)| - \langle c_i \rangle_t)\ . \qquad \text{(eq. S9)}$$

Here, $\langle c_i \rangle_t$ are the time-averaged MO coefficients. We applied a $\cos^2$-envelope function, which improved the signal-to-noise ratio with minimal broadening, followed by applying the fft.rfft() function from NumPy (https://numpy.org/) to obtain the power spectrum of $C_\Phi(\tau)$ .

7.4 Vibrational spectral density

Vibrational power spectral densities were calculated for NVE trajectories with TRAVIS.[13]

7.5 Radial and angular distribution functions

Radial distribution functions were constructed from the NVT trajectories employing the auto-FOX package (https://github.com/nlesc-nano/auto-FOX). Angular distribution functions of the Pb-Br-Pb angles were constructed with a custom-made code or using the auto-FOX package

7.6 Spatial correlation

QDs tumble during MD simulations, hence we first aligned the structures in each frame to a reference structure where the QD edges are parallel to the x, y and z axes. To calculate the spatial correlation, we first generate a rasterized 3D image of the Pb-Br sublattice by binning the Pb and Br positions and convoluting/filtering with a Gaussian of a width of 0.2 Angstrom. The images are then autocorrelated along the x-axis to obtain the spatial correlation.

7.7 Movie of HOMO wavefunction and time-averaged wavefunction.

The normalized wavefunction density was obtained from cube files of the HOMO wavefunction. To correct for tumbling of the QD during the simulations, matrix rotations by Euler angles that align the QD structure to a reference structure are applied to the cube (3D) image of the density. The density is then integrated along on axis to obtain the two-dimensional projections that are shown in the movie or time-integrated for the time-averaged representation of the wavefunction.

7.8 Inverse participation ratio

Using the cube files of the HOMO wavefunction ($\Psi(\boldsymbol{r})$) printed every 0.1 ps, the inverse participation ratio (IPR) of the HOMO wavefunction was computed from its probability density $\rho(\boldsymbol{r}) = |\Psi(\boldsymbol{r})|^2$ from

$$\mathrm{IPR} = \frac{\int \rho(\boldsymbol{r})^2 d\boldsymbol{r}}{(\int \rho(\boldsymbol{r}) d\boldsymbol{r})^2} . \qquad \text{(eq. S10)}$$

The IPR was then averaged along the AIMD trajectory.

**Supplementary Note 8: LUMO wavefunction localization**

Perovskite QD models without full surface passivation create surface-localized LUMOs (lowest unoccupied molecular orbitals) due to undercoordinated bromine atoms, see Supplementary Fig. 10a as well as previous works on the topic.[14,15] However, the electrostatic environment provided by surface ligands can be emulated by epitaxially shelling $CsPbBr_3$ with $CsCaBr_3$-shell, as shown in Supplementary Fig. 10b. This wide-bandgap shell enables the delocalization of the LUMO wavefunction across the QD core.[16,17] Employing such a surface passivation for $CsPbBr_3$ enables a full analysis of the LUMO wavefunction localization, which is summarized in Supplementary Fig 10. A shelling material that is dielectric and lattice-matched, like $CsCaBr_3$ for $CsPbBr_3$, is yet to be identified for $FAPbBr_3$. On the other hand, eliminating any surface effects, simulations under periodic boundary conditions offer a comparison of the LUMO wavefunction localization in $CsPbBr_3$ and $FAPbBr_3$, which is summarized in Supplementary Fig 11.

## Supplementary Figures

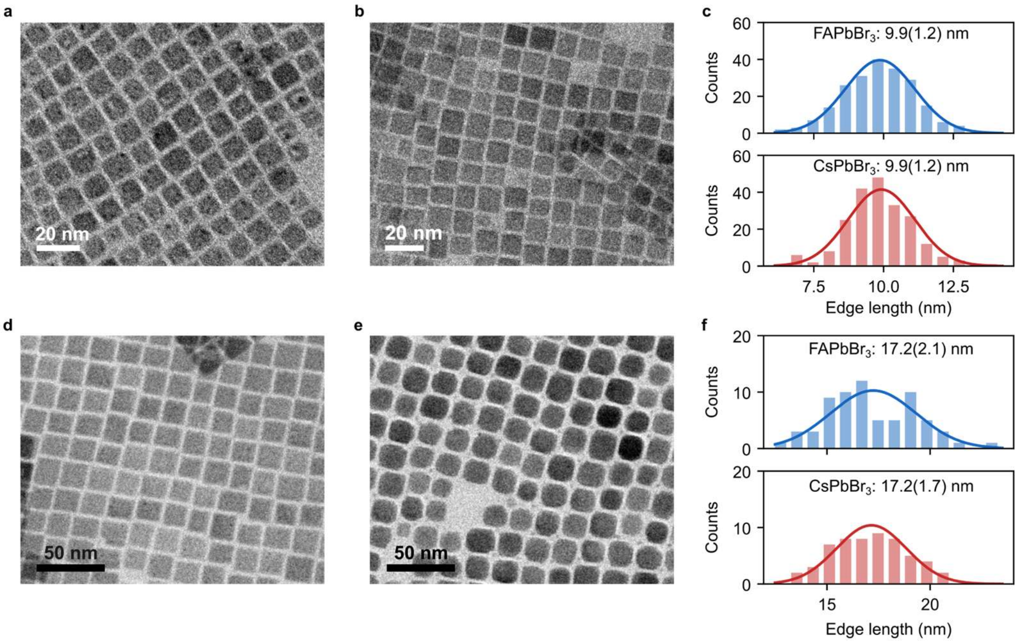


Supplementary Figure 1: QD-size determination for $FAPbBr_3$ and $CsPbBr_3$ QDs. **a**, TEM images of $FAPbBr_3$ QDs with a mean edge length of 9.9(1.2) nm. **b**, TEM images of $CsPbBr_3$ QDs with a mean edge length of 9.9(1.2) nm. **c**, Histograms of the measured edge lengths from samples in a and b. **d**, TEM images of $FAPbBr_3$ QDs with a mean edge length of 17.2(2.1) nm. **e**, TEM images of $CsPbBr_3$ QDs with a mean edge length of 17.2(1.7) nm. **f**, Histograms of the measured edge lengths from samples in d and e.

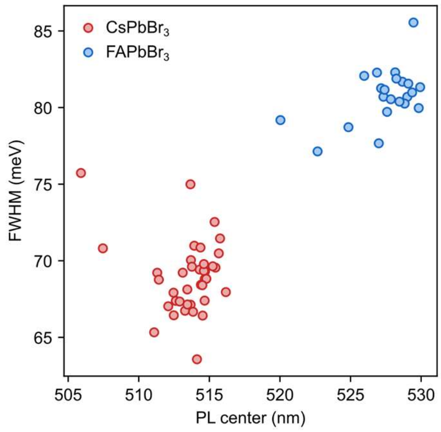


Supplementary Figure 2: Single-particle PL broadening (FWHM) at room temperature for $FAPbBr_3$ QDs with an edge length of 10.2(1.2) nm (blue markers) and $CsPbBr_3$ QDs with an edge length of 10.8(1.3) nm (red markers).

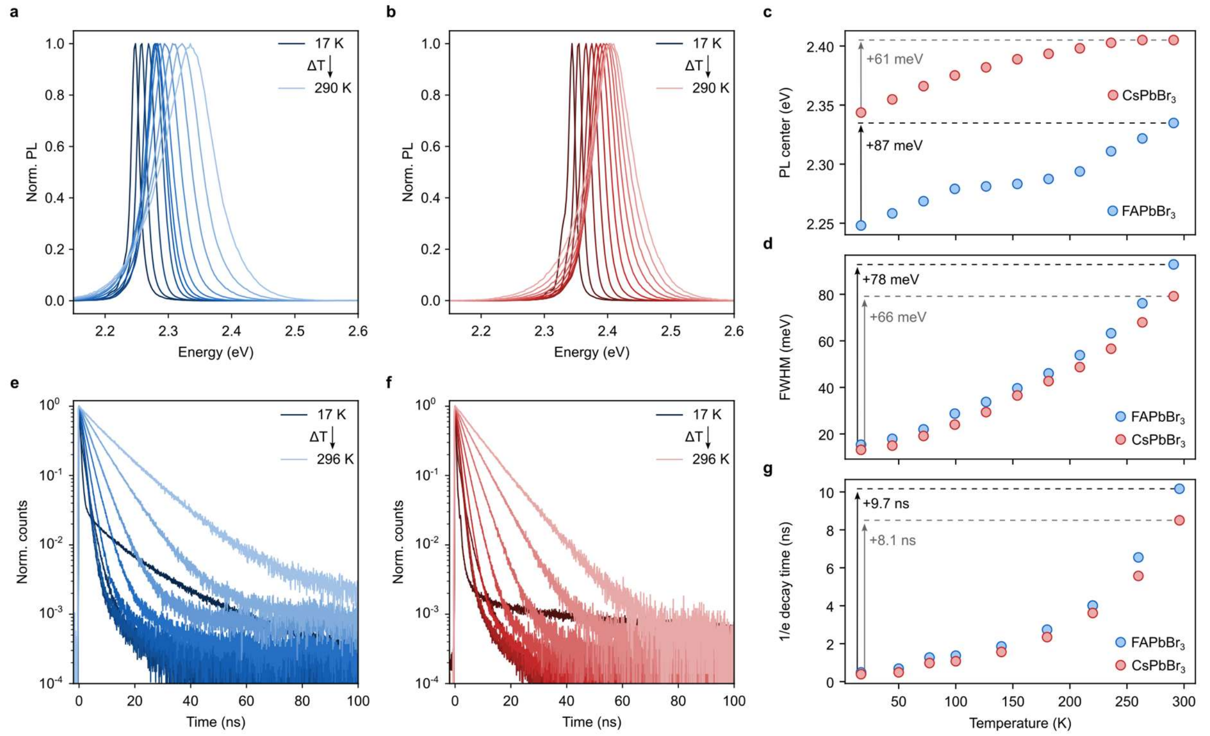


Supplementary Figure 3: Variable-temperature ensemble PL and TRPL measurements of $FAPbBr_3$ and $CsPbBr_3$ QD thin films from samples with edge lengths of 10.2(1.2) and 10.8(1.3) nm, respectively. **a,b,** PL spectra of $FAPbBr_3$ (a) and $CsPbBr_3$ (b) QDs as a function of temperature. **c,** PL peak center as a function of temperature extracted from the spectra in a and b. **d,** PL peak width (FWHM) extracted from spectra in a and b. **e,f,** TRPL traces of $FAPbBr_3$ (e) and $CsPbBr_3$ (f) QD thin films as a function of temperature. **g,** 1/e decay times extracted from the traces in e and f.

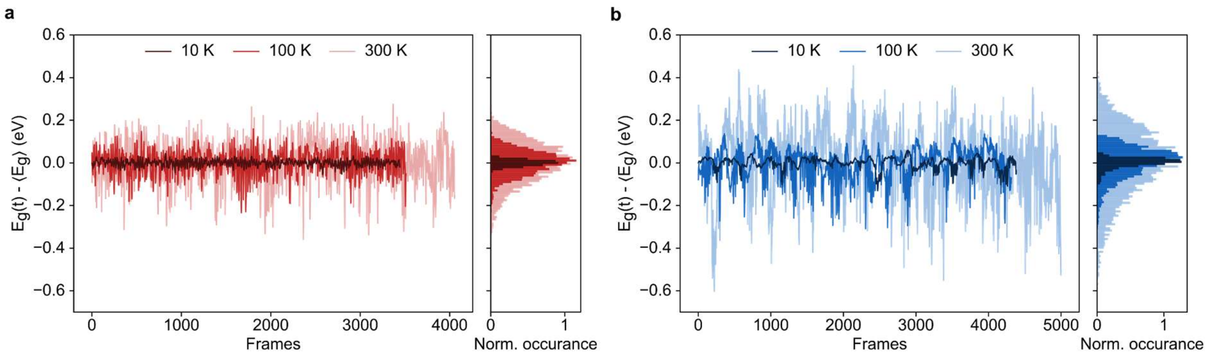


Supplementary Figure 4: Bandgap ($E_g$) during AIMD simulations of a 3.6 nm QD in the microcanonical ensemble. **a,** Bandgap fluctuation of a $CsPbBr_3$ QD at 10, 100, and 300 K. **b,** Bandgap fluctuation of a $FAPbBr_3$ QD at 10, 100, and 300 K.

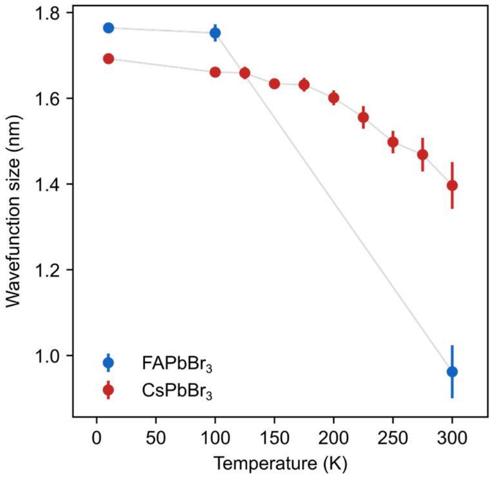


Supplementary Figure 5: Temperature dependence of the HOMO wavefunction size in 3.6 nm QDs. Average HOMO wavefunction sizes (markers) and 95% confidence intervals (error bars) for a $FAPbBr_3$ QD (blue) and a $CsPbBr_3$ QD (red) between 10 K and 300 K.

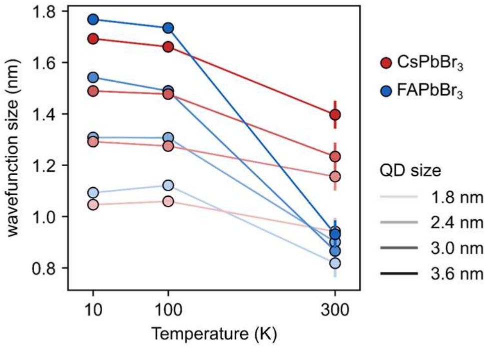


Supplementary Figure 6: Temperature dependence of HOMO wavefunction sizes extracted from AIMD simulations of $CsPbBr_3$ (red) and $FAPbBr_3$ QDs (blue) with different particle sizes. In $FAPbBr_3$, wavefunction sizes are nearly size-independent at 300 K. Error bars indicate 95% confidence intervals.

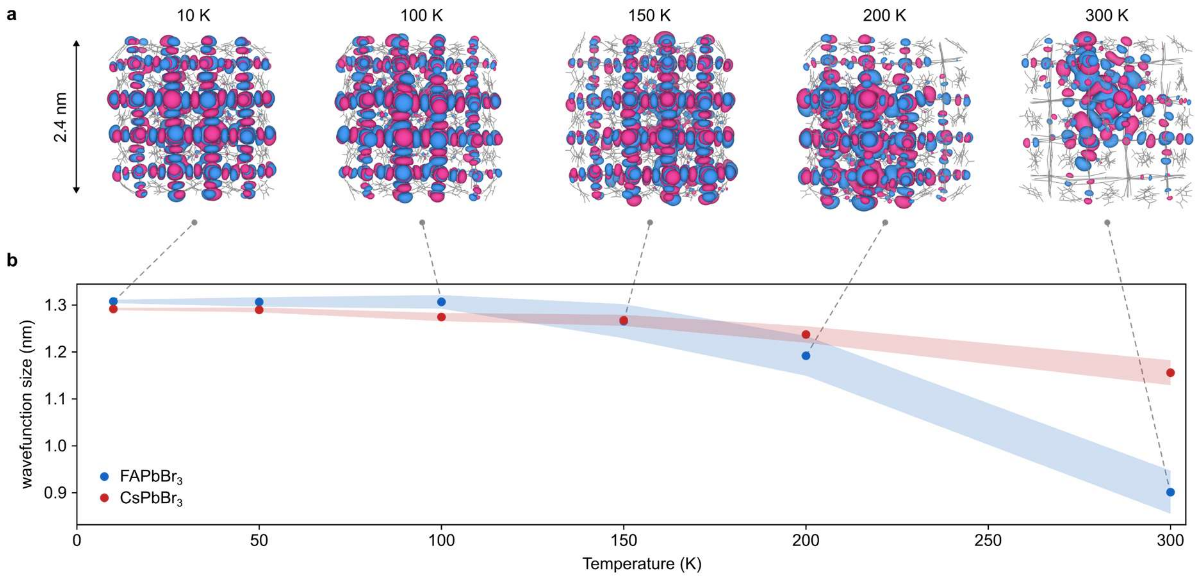


Supplementary Figure 7: Temperature dependence of the HOMO wavefunction size of QDs with edge lengths of 2.4 nm. **a,** Representative snapshots of the HOMO wavefunction in a $FAPbBr_3$ QD during AIMD trajectories at various temperatures between 10 K and 300 K. **b,** Average HOMO wavefunction sizes (markers) and 95% confidence intervals (shaded area) for a $FAPbBr_3$ QD (blue) and a $CsPbBr_3$ QD (red) between 10 K and 300 K.

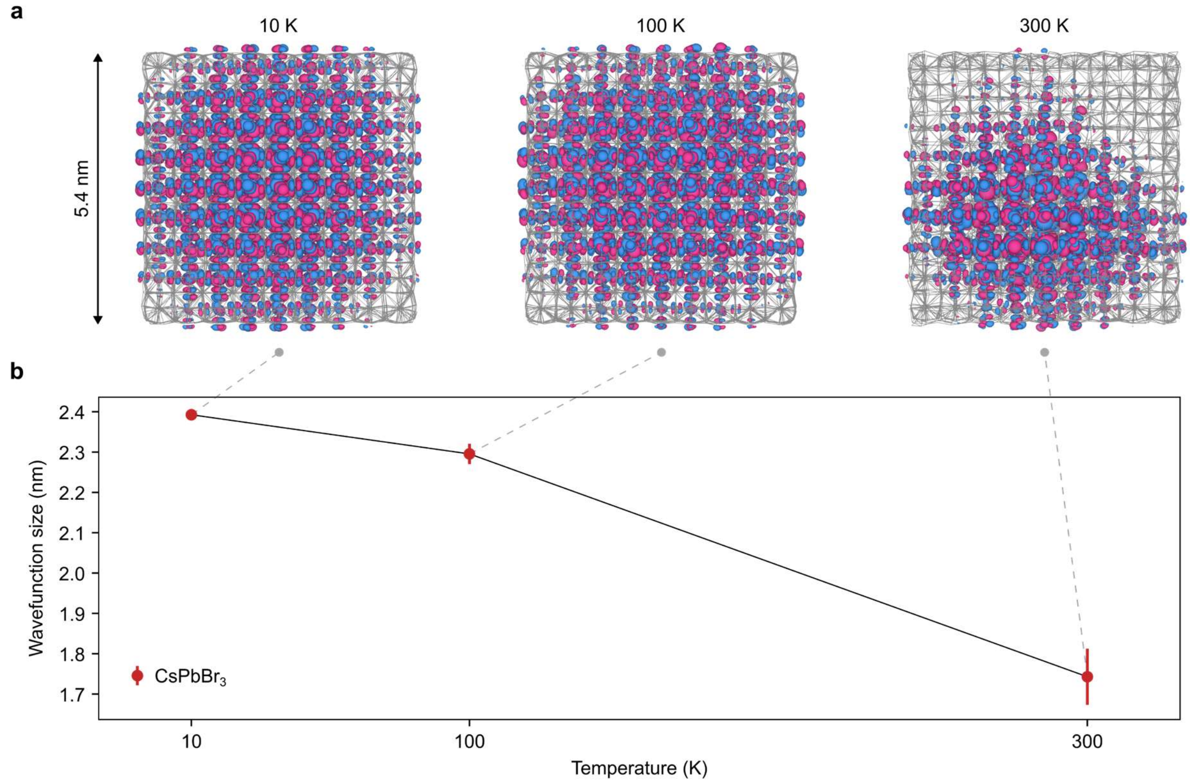


Supplementary Figure 8: Temperature-induced HOMO wavefunction localization in AIMD simulations of a 5.4 nm $CsPbBr_3$ QD model. **a,** Representative HOMO wavefunctions at 10, 100 and 300 K. **b,** Mean HOMO wavefunction size as a function of temperature. Error bars indicate 95% confidence intervals.

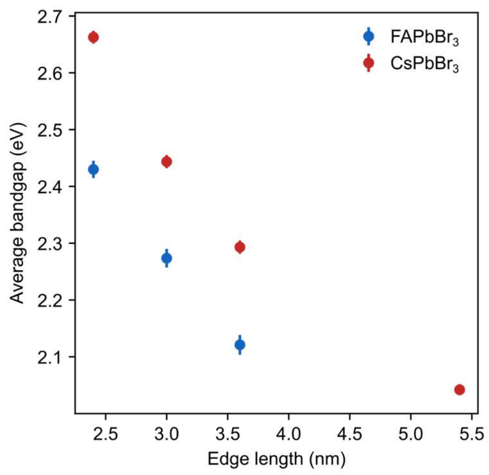


Supplementary Figure 9: Average bandgap of $FAPbBr_3$ and $CsPbBr_3$ QDs in AIMD simulations at 300 K as a function of their edge length. Error bars indicate 95% confidence intervals.

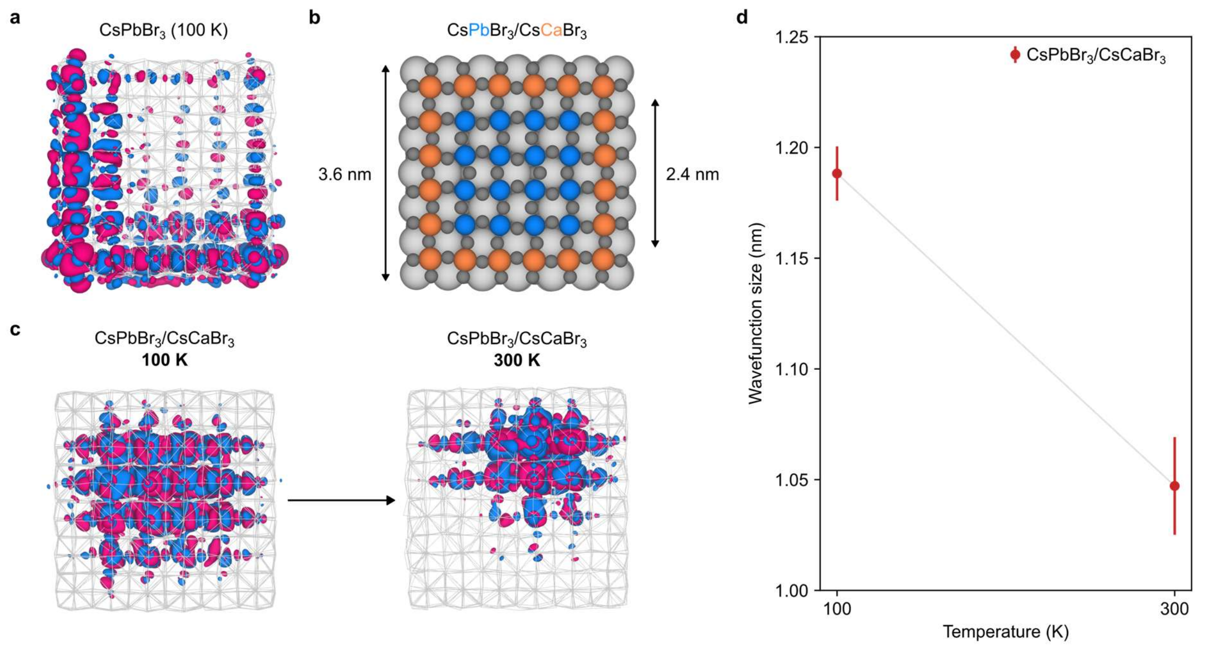


Supplementary Figure 10: Temperature-induced LUMO wavefunction localization in AIMD simulations of a 3.6 nm $CsPbBr_3/CsCaBr_3$ core/shell QD. **a,** Representative LUMO wavefunction in a 3.6 nm $CsPbBr_3$ QD at 100 K. **b,** Cross-section of a 3.6 nm $CsPbBr_3/CsCaBr_3$ core/shell QD with a 2.4 nm $CsPbBr_3$ core. **c,** Representative LUMO wavefunctions of a 3.6 nm $CsPbBr_3/CsCaBr_3$ core/shell QD at 100 and 300 K. **d,** Average LUMO wavefunction sizes (full width at half maximum, markers) and 95% confidence intervals (error bars) at 100 K and 300 K.

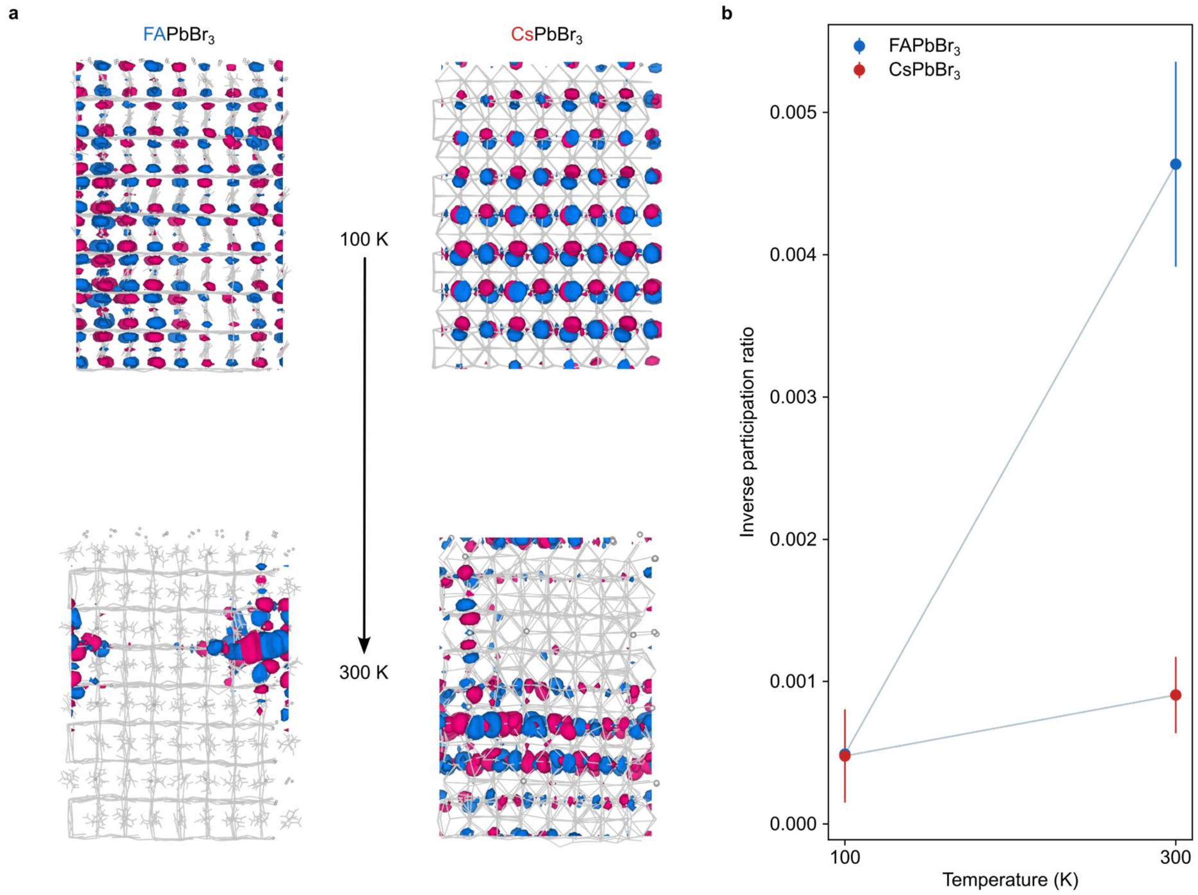


Supplementary Figure 11: Temperature-induced LUMO wavefunction localization in AIMD simulations of bulk $FAPbBr_3$ and $CsPbBr_3$. **a,** Representative LUMO wavefunctions at 100 and 300 K. **b,** Mean inverse participation ratio (IPR) as a function of temperature. Error bars indicate 95% confidence intervals.

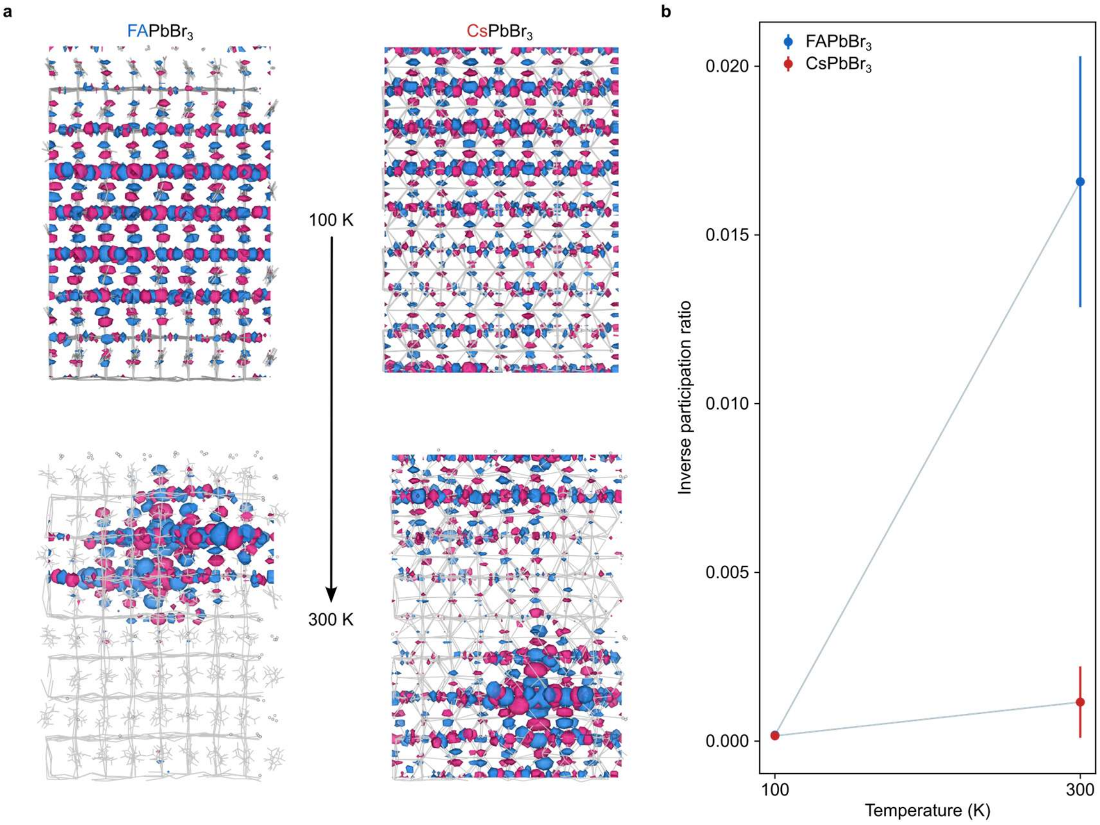


Supplementary Figure 12: Temperature-induced HOMO wavefunction localization in AIMD simulations of bulk $FAPbBr_3$ and $CsPbBr_3$. **a,** Representative HOMO wavefunctions at 100 and 300 K. **b,** Mean inverse participation ratio (IPR) as a function of temperature. Error bars indicate 95% confidence intervals.

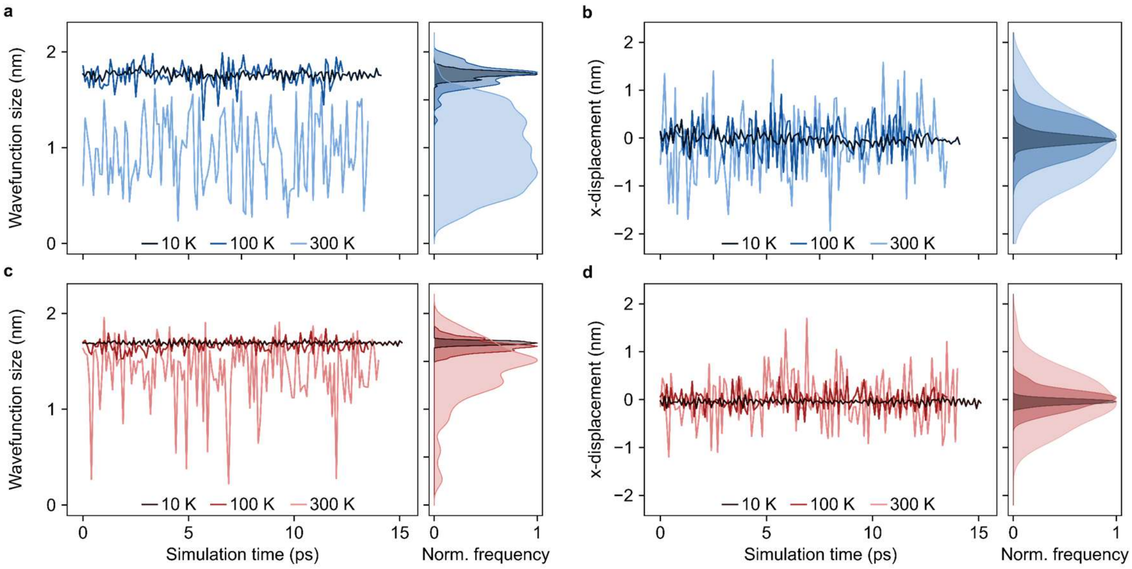


Supplementary Figure 13: Dynamic wavefunction localization in NVT AIMD simulations at 10, 100 and 300 K, for 3.6 nm QDs. **a**, HOMO wavefunction size as a function of simulation time (left) and corresponding histogram (right) of a 3.6 nm $FAPbBr_3$ QD. **b**, Displacement of the HOMO wavefunction

density's center-of-mass (COM) from the QD's COM along the x-direction as function of simulation time (left) and corresponding histogram (right) of a 3.6 nm $FAPbBr_3$ QD. **c,d**, Same as (a,b) but for a 3.6 nm $CsPbBr_3$ QD.

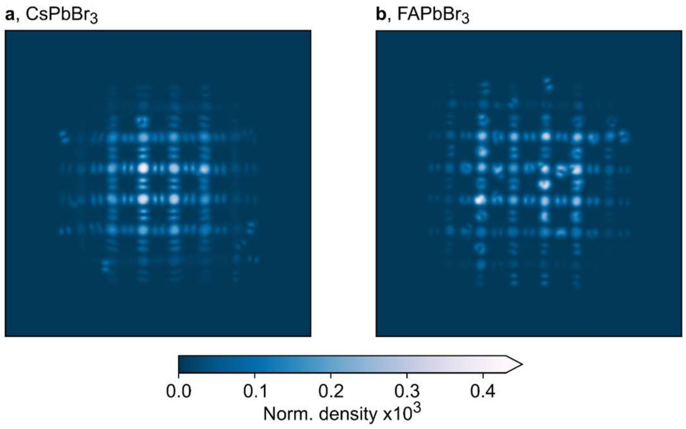


Supplementary Figure 14: Time-average HOMO wavefunction density of a $CsPbBr_3$ QD (a) and a $FAPbBr_3$ QD (b) with edge lengths of 3.6 nm in 300 K NVT AIMD simulations, integrated along the z-direction.

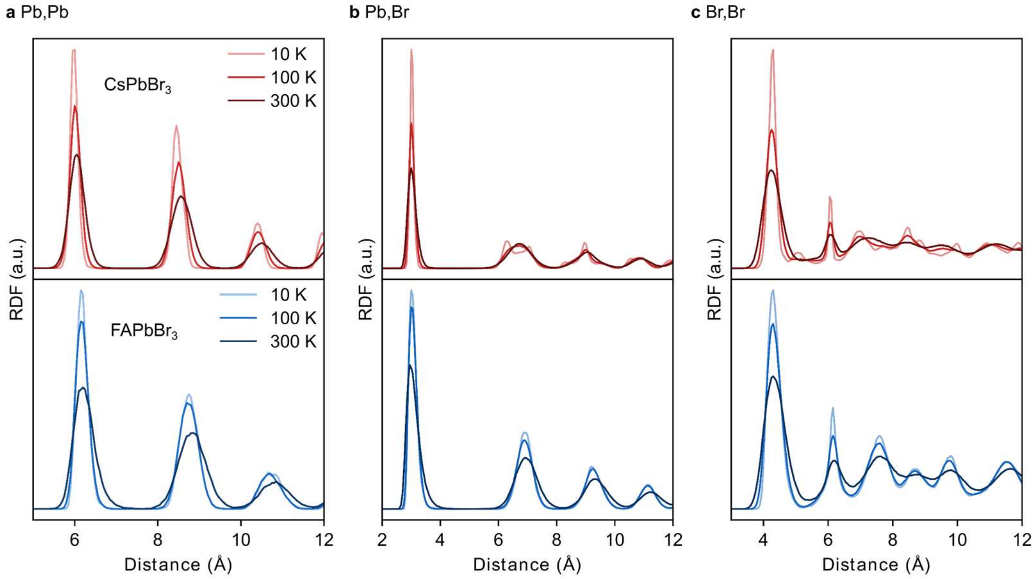


Supplementary Figure 15: Temperature-dependence of radial distribution functions (RDFs) of different atom pairs in the Pb-Br sublattice from AIMD simulations of $CsPbBr_3$ (top row, red) and $FAPbBr_3$ QDs (bottom row, blue) with edge lengths of 3.6 nm. The broadness of the distributions indicates disorder. Note that the vertical axes change from one row to another.

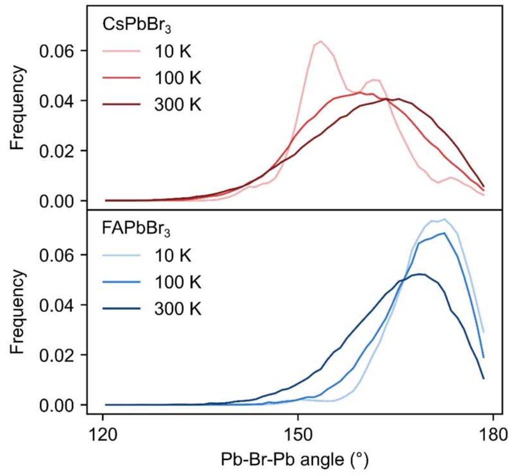


Supplementary Figure 16: Temperature-dependence of angular distribution functions of Pb-Br-Pb angles in AIMD simulations of $CsPbBr_3$ (top row, red) and $FAPbBr_3$ QDs (bottom row, blue) with edge lengths of 3.6 nm.

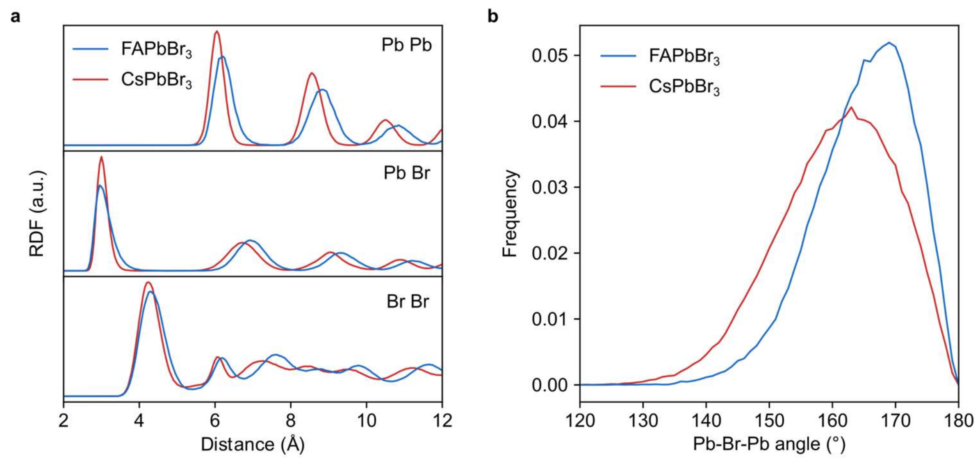


Supplementary Figure 17: Comparison of structural descriptors of the Pb-Br framework in 3.6 nm models of $FAPbBr_3$ and $CsPbBr_3$ QDs at 300 K. **a,** Radial distribution functions. **b,** Histogram of Pb-Br-Pb angles measuring octahedral tilting.

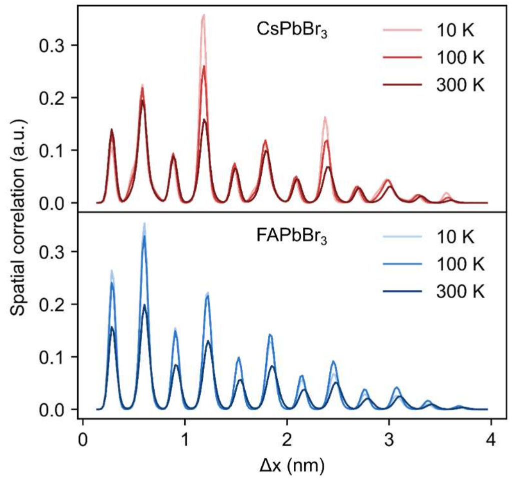

Supplementary Figure 18: Temperature-dependence of spatial correlations in the Pb-Br sublattice in AIMD simulations of $CsPbBr_3$ (top, red) and $FAPbBr_3$ QDs (bottom, blue) with edge lengths of 3.6 nm.

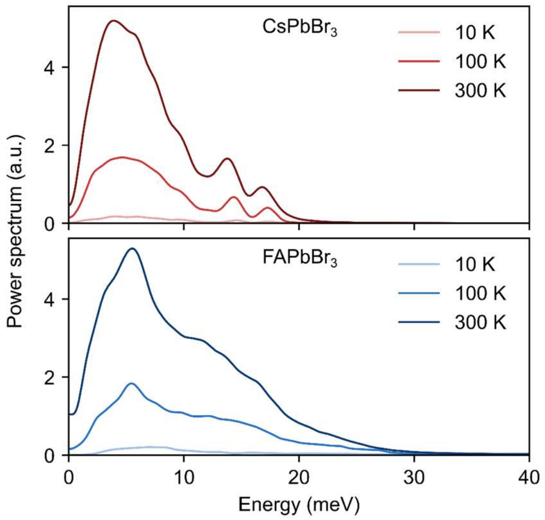


Supplementary Figure 19: Temperature-dependence of the vibrational spectral density in AIMD simulations of $CsPbBr_3$ (top row, red) and $FAPbBr_3$ QDs (bottom row, blue) with edge lengths of 2.4 nm.

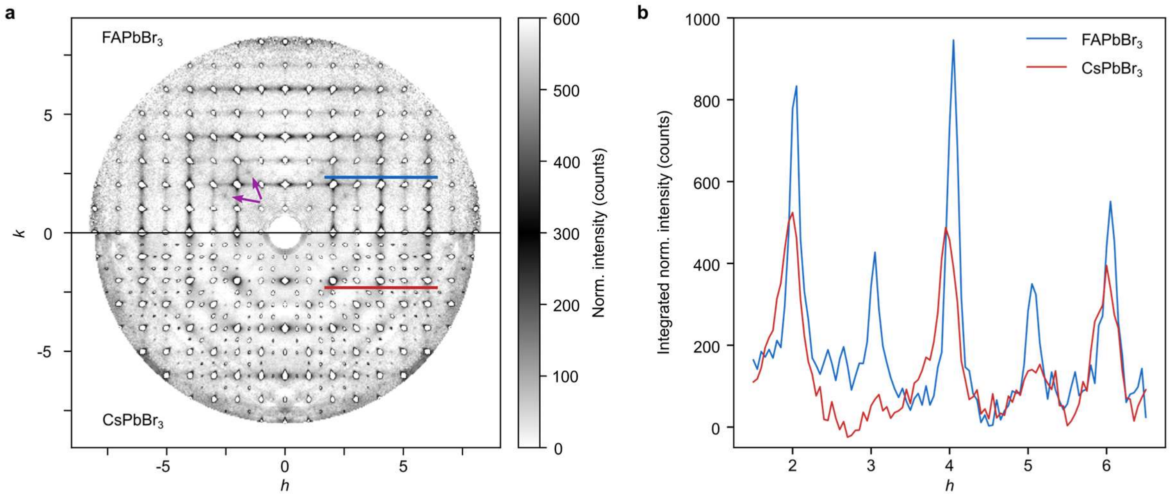


Supplementary Figure 20: Normalized X-ray total scattering of $FAPbBr_3$ and $CsPbBr_3$ single crystals at room temperature. **a,** Total scattering pattern in the *hk*0 plane of $FAPbBr_3$ (top) and $CsPbBr_3$ (bottom). Purple arrows indicate the local symmetry breaking peaks in $FAPbBr_3$. **b,** Profile along *h*(2.25)0 (lines indicated in a) highlighting the diffuse scattering in $FAPbBr_3$ (blue) and $CsPbBr_3$ (red).

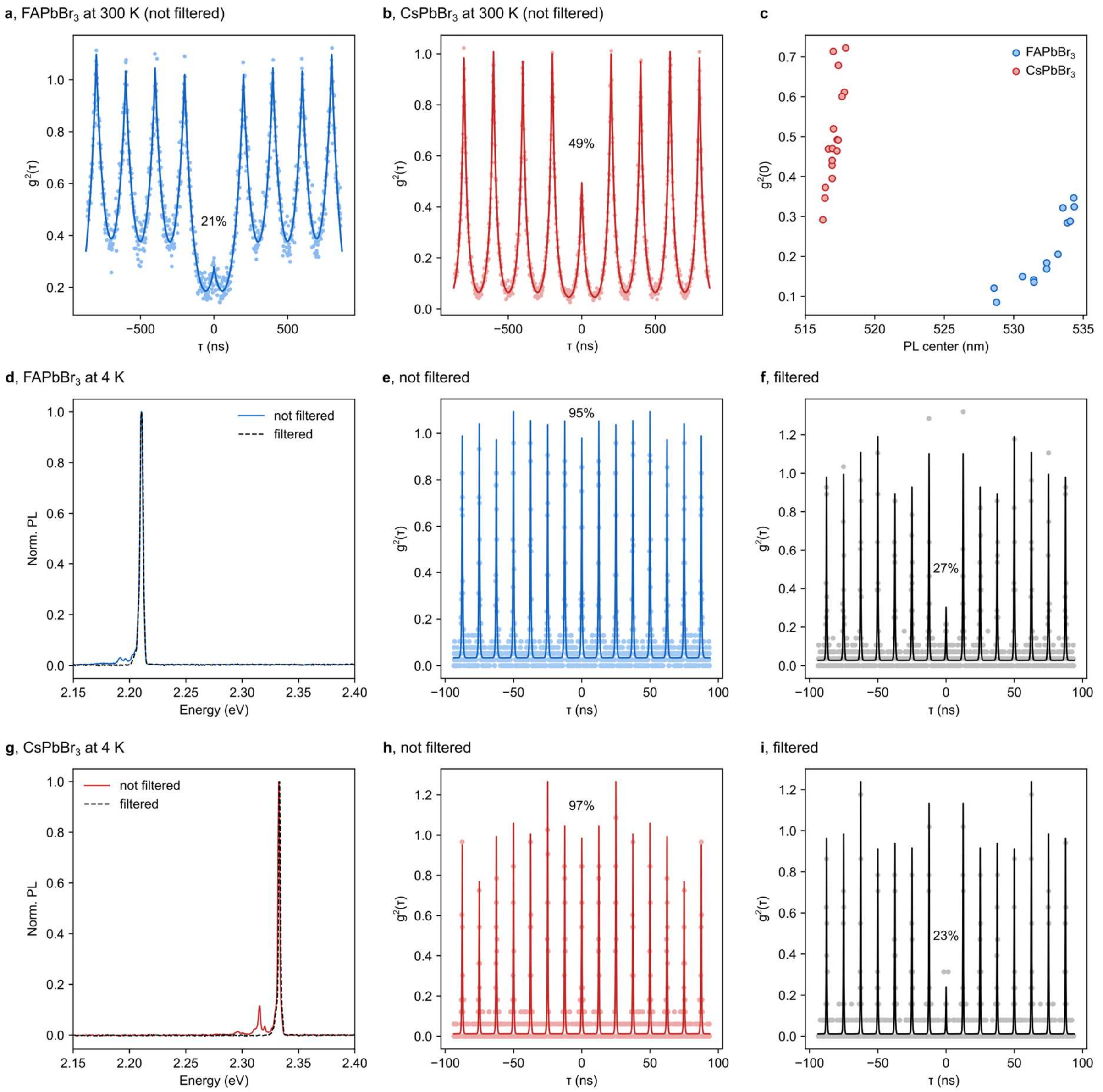


Supplementary Figure 21: Single-particle PL measurements of QDs with 17.2 nm edge lengths at room temperature and 4 K. **a,** Second-order correlation function, $g^2(\tau)$, of a representative $FAPbBr_3$ QD at room temperature. **b,** $g^2(\tau)$ of a representative $CsPbBr_3$ QD at room temperature. **c,** Histogram of $g^2(0)$ values of $FAPbBr_3$ QDs (blue markers) with an edge length of 17.2(1.7) nm and $CsPbBr_3$ QDs (red markers) with an edge length of 17.2(2.1) nm. **d,** Spectrum of a single $FAPbBr_3$ QD obtained at 4 K (solid line). A tunable spectral filter is used to retain only single excitons (dashed line). **e,** $g^2(\tau)$ of the $FAPbBr_3$ QD obtained at 4 K. **f,** $g^2(\tau)$ of the $FAPbBr_3$ QD at 4 K, obtained upon spectral filtering. **g,** Spectrum of a single $CsPbBr_3$ QD obtained at 4 K (solid line). A tunable spectral filter is used to retain only the single excitons (dashed line). **h,** $g^2(\tau)$ of the $CsPbBr_3$ QD obtained at 4 K. **i,** $g^2(\tau)$ of the $CsPbBr_3$ QD at 4 K, obtained upon spectral filtering.

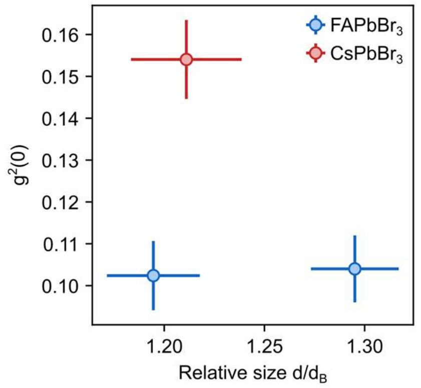


Supplementary Figure 22: Comparison of single-photon purity of $FAPbBr_3$ and $CsPbBr_3$ QDs (markers) and 95% confidence intervals (error bars) at identical relative sizes.

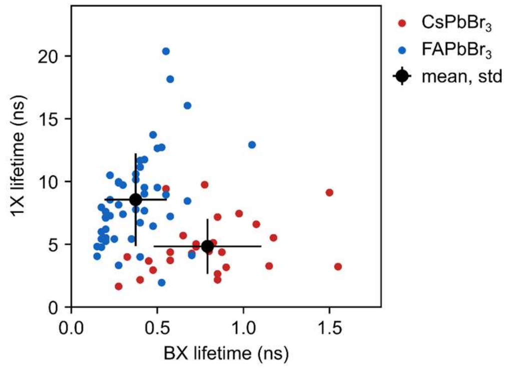


Supplementary Figure 23: Biexciton and exciton excited state lifetimes of $FAPbBr_3$ and $CsPbBr_3$ QDs probed by heralded spectroscopy.

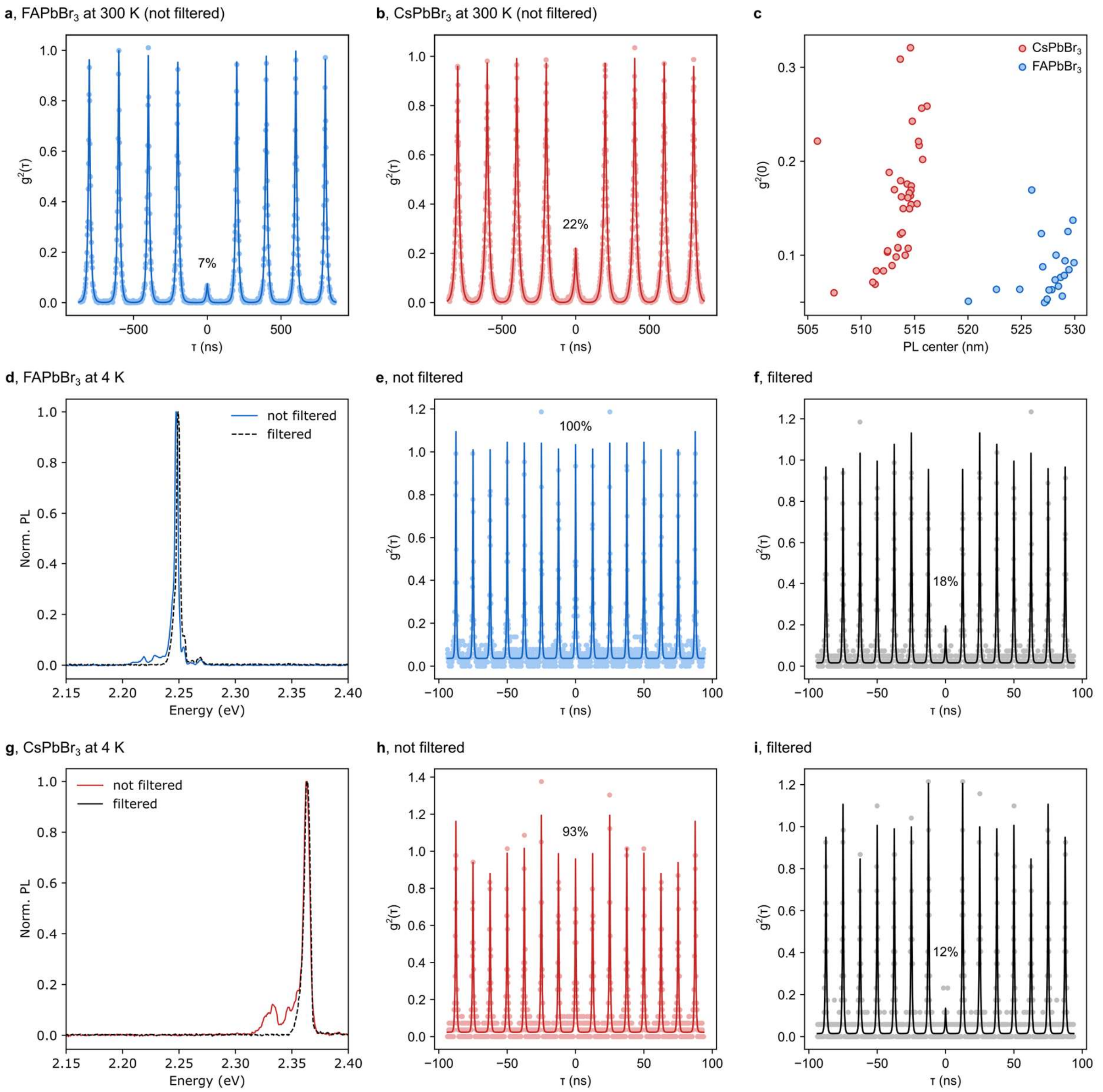


Supplementary Figure 24: Single-particle PL measurements of QDs with 10 nm edge lengths at room temperature and 4 K. **a,** Second-order correlation function, $g^2(\tau)$, of a representative $FAPbBr_3$ QD at room temperature. **b,** $g^2(\tau)$ of a representative $CsPbBr_3$ QD at room temperature. **c,** Histogram of $g^2(0)$ values of $FAPbBr_3$ QDs (blue markers) with an edge length of 10.2(1.2) nm and $CsPbBr_3$ QDs (red markers) with an edge length of 10.8(1.3) nm. **d,** Spectrum of a single $FAPbBr_3$ QD obtained at 4 K (solid line). A tunable spectral filter is used to retain only single excitons (dashed line). **e,** $g^2(\tau)$ of the $FAPbBr_3$ QD obtained at 4 K. **f,** $g^2(\tau)$ of the $FAPbBr_3$ QD at 4 K, obtained upon spectral filtering. **g,** Spectrum of a single $CsPbBr_3$ QD obtained at 4 K (solid line). A tunable spectral filter is used to retain only the single excitons (dashed line). **h,** $g^2(\tau)$ of the $CsPbBr_3$ QD obtained at 4 K. **i,** $g^2(\tau)$ of the $CsPbBr_3$ QD at 4 K, obtained upon spectral filtering.

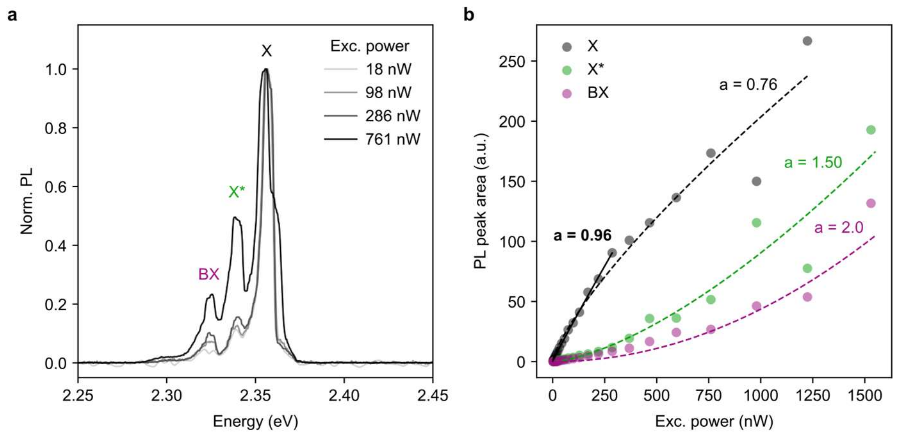


Supplementary Figure 25: Power dependence of the PL spectrum of a single $CsPbBr_3$ QD recorded at 4 K. **a,** Single-particle PL spectrum at varying laser power featuring exciton (X), trion (X*), and biexciton (BX) species, with intensities normalized to the exciton peak. **b,** Excitation-power dependence of the PL peak area of the three species in panel a (markers). Power-law fits providing exponentials of 0.76 (0.95 at low power) for the exciton, 1.50 for the trion and 2.0 for the biexciton emission reinforce the assignment of the species in the spectrum.

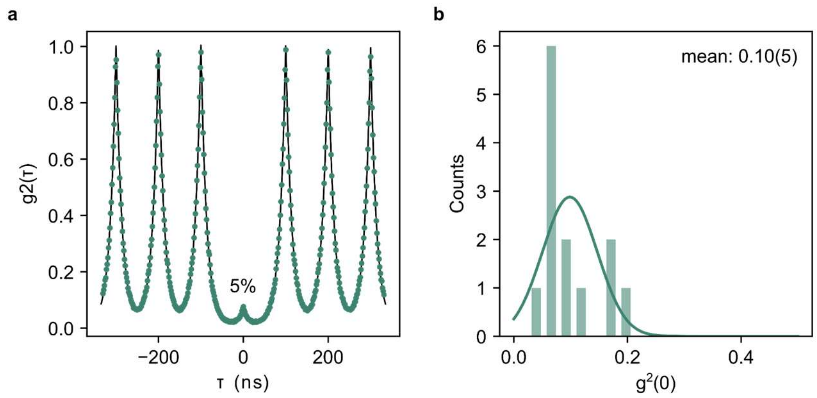


Supplementary Figure 26: Single-photon purity of weakly confined $MAPbBr_3$ QDs at room temperature. Representative $g^2(\tau)$ (a) and histogram of $g^2(0)$ (b) from as sample of $MAPbBr_3$ QDs with an edge length of 9.4(1.4) nm.

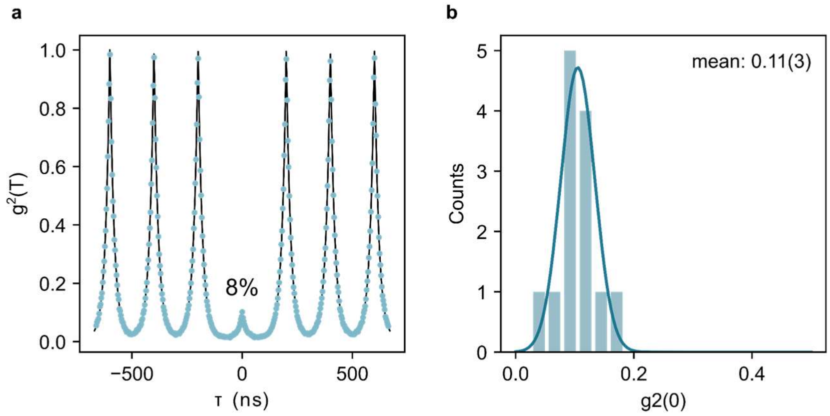

Supplementary Figure 27: Single-photon purity of weakly confined AZPbBr$_3$ QDs at room temperature. Representative $g^2(\tau)$ (a) and histogram of $g^2(0)$ (b) from as sample of AZPbBr$_3$ QDs with an edge length of 10.4(1.3) nm.

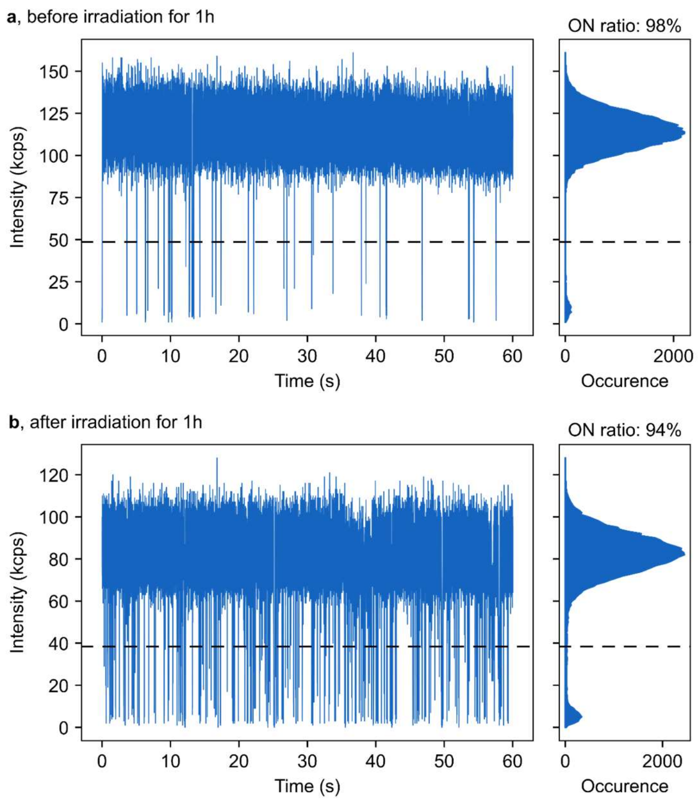


Supplementary Figure 28: Blinking of a FAPbBr$_3$ QD before (a) and after (b) continuous irradiation for 1h. Intensity time traces were constructed with a bin width of 1 ms.

## References

1 Morad, V. *et al.* Designer phospholipid capping ligands for soft metal halide nanocrystals. *Nature* **626**, 542-548 (2024).
2 Papagiorgis, P. *et al.* Unraveling the Radiative Pathways of Hot Carriers upon Intense Photoexcitation of Lead Halide Perovskite Nanocrystals. *ACS Nano* **13**, 5799-5809 (2019).
3 Bodnarchuk, M. I. *et al.* Colloidal Aziridinium Lead Bromide Quantum Dots. *ACS Nano* **18**, 5684-5697 (2024).
4 Dolomanov, O. V., Bourhis, L. J., Gildea, R. J., Howard, J. A. K. & Puschmann, H. OLEX2: a complete structure solution, refinement and analysis program. *J. Appl. Crystallogr.* **42**, 339-341 (2009).
5 Sheldrick, G. M. SHELXT - integrated space-group and crystal-structure determination. *Acta Crystallogr., Sect. A: Found. Adv.* **71**, 3-8 (2015).
6 Sheldrick, G. M. Crystal structure refinement with SHELXL. *Acta Crystallogr., Sect. C: Struct. Chem.* **71**, 3-8 (2015).
7 Momma, K. & Izumi, F. VESTA 3for three-dimensional visualization of crystal, volumetric and morphology data. *J. Appl. Crystallogr.* **44**, 1272-1276 (2011).
8 Kabsch, W. Xds. *Acta Crystallogr., Sect. D:Biol. Crystallogr.* **66**, 125-132 (2010).
9 D'Amato, M. *et al.* Highly Photostable Zn-Treated Halide Perovskite Nanocrystals for Efficient Single Photon Generation. *Nano Lett.* **23**, 10228-10235 (2023).
10 Lubin, G. *et al.* Heralded Spectroscopy Reveals Exciton-Exciton Correlations in Single Colloidal Quantum Dots. *Nano Lett.* **21**, 6756-6763 (2021).
11 Frenkel, N. *et al.* Two Biexciton Types Coexisting in Coupled Quantum Dot Molecules. *ACS Nano* **17**, 14990-15000 (2023).
12 Lubin, G. *et al.* Quantum correlation measurement with single photon avalanche diode arrays. *Opt. Express* **27**, 32863-32882 (2019).
13 Brehm, M., Thomas, M., Gehrke, S. & Kirchner, B. TRAVIS-A free analyzer for trajectories from molecular simulation. *J. Chem. Phys.* **152**, 164105 (2020).
14 du Fosse, I. *et al.* Limits of Defect Tolerance in Perovskite Nanocrystals: Effect of Local Electrostatic Potential on Trap States. *J. Am. Chem. Soc.* **144**, 11059-11063 (2022).
15 Bodnarchuk, M. I. *et al.* Rationalizing and Controlling the Surface Structure and Electronic Passivation of Cesium Lead Halide Nanocrystals. *ACS Energy Lett.* **4**, 63-74 (2019).
16 Raino, G. *et al.* Ultra-narrow room-temperature emission from single $CsPbBr_3$ perovskite quantum dots. *Nat. Commun.* **13**, 2587 (2022).
17 Zhu, C. *et al.* Single-photon superradiance in individual caesium lead halide quantum dots. *Nature* **626**, 535-541 (2024).

| | |
|---|---|
| Filename: | SUPPLEMENTARY_INFORMATION.docx |
| Directory: | C:\Users\leong\Documents |
| Template: | C:\Users\leong\AppData\Roaming\Microsoft\Templates\Normal.dotm |
| Title: | |
| Subject: | |
| Author: | Feld Leon |
| Keywords: | |
| Comments: | |
| Creation Date: | 11/3/2025 11:23:00 AM |
| Change Number: | 22 |
| Last Saved On: | 12/17/2025 9:34:00 AM |
| Last Saved By: | Leon Feld |
| Last Printed On: | 12/17/2025 9:34:00 AM |

As of Last Complete Printing

| | |
|---|---|
| Number of Pages: | 29 |
| Number of Words: | 8’808 (approx.) |
| Number of Characters: | 50’212 (approx.) |
