# Supplementary material for: Phonon-driven wavefunction localization enhances room-temperature single-photon purity in large hybrid lead halide perovskite quantum dots
Source: Nat Commun. 2026 Jan 23;17:1974. doi: 10.1038/s41467-026-68607-w (PMC12932643; doi:10.1038/s41467-026-68607-w)
Supplement: Supplementary file 2 — Description of Additional Supplementary Files [file 41467_2026_68607_MOESM2_ESM.pdf]

File Name: SupplementaryData1_cif_checkcif_tables.zip
A folder containing crystal structure solutions of $CsPbBr_3$ (Cambridge Structural Database entry 2407795, CIF file and HTML file) and $FAPbBr_3$ (Cambridge Structural Database entry 2407691, CIF file and HTML file) single crystals, along with the respective Checkcif files (in PDF).

File Name: SupplementaryVideo1.mp4
Description: Movie of HOMO wavefunction density projections for a 3.6 nm $CsPbBr_3$ QD along an MD trajectory at 300 K.

File Name: SupplementaryVideo2.mp4
Description: Movie of HOMO wavefunction density projections for a 3.6 nm $FAPbBr_3$ QD along an MD trajectory at 300 K.

File Name: SupplementaryVideo3.mp4
Description: Movie of HOMO wavefunction density projections for a 5.4 nm $CsPbBr_3$ QD along an MD trajectory at 300 K.

File Name: SupplementaryVideo4.mp4
Description: Movie of HOMO wavefunction density projections for bulk $CsPbBr_3$ along an MD trajectory at 300 K.

File Name: SupplementaryVideo5.mp4
Description: Movie of HOMO wavefunction density projections for bulk $FAPbBr_3$ along an MD trajectory at 300 K.

File Name: SupplementaryVideo6.mp4
Description: Movie of LUMO wavefunction density projections for bulk $CsPbBr_3$ along an MD trajectory at 300 K.

File Name: SupplementaryVideo7.mp4
Description: Movie of LUMO wavefunction density projections for bulk $FAPbBr_3$ along an MD trajectory at 300 K.

File Name: SupplementaryVideo8.mp4
Description: Movie of LUMO wavefunction density projections for a 3.6 nm $CsPbBr_3$/$CsCaBr_3$ core/shell QD along an MD trajectory at 300 K.
