# Supplementary material for: Phonon-driven wavefunction localization enhances room-temperature single-photon purity in large hybrid lead halide perovskite quantum dots
Source: Nat Commun. 2026 Jan 23;17:1974. doi: 10.1038/s41467-026-68607-w (PMC12932643; doi:10.1038/s41467-026-68607-w)
Supplement: Supplementary file 3 — Supplementary Data 1 [file 41467_2026_68607_MOESM3_ESM.zip › 2407691_Checkcif.pdf]

No syntax errors found.
Please wait while processing ....

CIF dictionary
Interpreting this report

# Datablock: FAPbBr3

| | | | |
|---|---|---|---|
| Bond precision: | Pb-Br = 0.0001 A | | Wavelength=0.71073 |
| Cell: | a=5.9990(1) | b=5.9990(1) | c=5.9990(1) |
| | alpha=90 | beta=90 | gamma=90 |
| Temperature: | 300 K | | |

| | Calculated | Reported |
|---|---|---|
| Volume | 215.892(11) | 215.892(11) |
| Space group | P m -3 m | P m -3 m |
| Hall group | -P 4 2 3 | -P 4 2 3 |
| Moiety formula | 3(Br Pb0.33), C H3 N2, 2(H) | Br3 Pb, 2(C0.5 H2.5 N) |
| Sum formula | C H5 Br3 N2 Pb | C H5 Br3 N2 Pb |
| Mr | 491.96 | 491.99 |
| Dx,g cm-3 | 3.784 | 3.784 |
| Z | 1 | 1 |
| Mu (mm-1) | 33.329 | 33.329 |
| F000 | 212.0 | 212.0 |
| F000' | 208.40 | |
| h,k,lmax | 8,8,8 | 8,8,8 |
| Nref | 90 | 88 |
| Tmin,Tmax | 0.121,0.189 | 0.681,1.000 |
| Tmin' | 0.078 | |

Correction method= # Reported T Limits: Tmin=0.681 Tmax=1.000
AbsCorr = MULTI-SCAN

Data completeness= 0.978 Theta(max)= 29.476

R(reflections)= 0.0089( 88) wR2(reflections)= 0.0228( 88)

S = 1.181 Npar= 10

The following ALERTS were generated. Each ALERT has the format
**test-name_ALERT_alert-type_alert-level**.
Click on the hyperlinks for more details of the test.

## Alert level C

```
PLAT042_ALERT_1_C Calc. and Reported MoietyFormula Strings  Differ     Please Check
            Calc: 3(Br Pb0.33), C H3 N2, 2(H)
            Rep.: Br3 Pb, 2(C0.5 H2.5 N)
PLAT088_ALERT_3_C Poor Data / Parameter Ratio ....................       8.80 Note
PLAT245_ALERT_2_C U(iso) H1        Smaller than U(eq) C1        by      0.035 Ang**2
PLAT260_ALERT_2_C Large Average Ueq of Residue Including        C1      0.131 Check
```

## Alert level G

```
PLAT002_ALERT_2_G Number of Distance or Angle Restraints on AtSite          5 Note
PLAT004_ALERT_5_G Polymeric Structure Found with Maximum Dimension          3 Info
PLAT007_ALERT_5_G Number of Unrefined Donor-H Atoms ..............          1 Report
            H1B
PLAT012_ALERT_1_G N.O.K.   _shelx_res_checksum Found in CIF ......     Please Check
PLAT013_ALERT_1_G N.O.K.   _shelx_hkl_checksum Found in CIF ......     Please Check
PLAT172_ALERT_4_G The CIF-Embedded .res File Contains DFIX Records          3 Report
PLAT173_ALERT_4_G The CIF-Embedded .res File Contains DANG Records          4 Report
PLAT174_ALERT_4_G The CIF-Embedded .res File Contains FLAT Records          1 Report
PLAT176_ALERT_4_G The CIF-Embedded .res File Contains SADI Records          1 Report
PLAT187_ALERT_4_G The CIF-Embedded .res File Contains RIGU Records          1 Report
PLAT190_ALERT_3_G A Non-default RIGU Restraint Value for First Par     1.4000 Report
PLAT190_ALERT_3_G A Non-default RIGU Restraint Value for SecondPar     1.4000 Report
PLAT232_ALERT_2_G Hirshfeld Test Diff (M-X)  Pb1      --Br1      .       10.8 s.u.
PLAT300_ALERT_4_G Atom Site Occupancy of N1          Constrained at     0.0833 Check
```

**And 3 other PLAT300 Alerts**
More ...

```
PLAT301_ALERT_3_G Main Residue  Disorder .............(Resd    2)          8% Note
PLAT764_ALERT_4_G Overcomplete CIF Bond List Detected (Rep/Expd) .       4.14 Ratio
```

```
PLAT779_ALERT_4_G Suspect or Irrelevant (Bond) Angle(s) in CIF ...      26.80 Deg.
              N1   -C1   -N1     27_555   1_555  17_555 ......     #   23 Check
And 28 other PLAT779 Alerts
More ...
PLAT860_ALERT_3_G Number of Least-Squares Restraints .............         11 Note
PLAT912_ALERT_4_G Missing # of FCF Reflections Above STh/L=  0.600          1 Note
PLAT933_ALERT_2_G Number of HKL-OMIT Records in Embedded .res File          2 Note
                0  0  8,   1  1  1,
PLAT940_ALERT_3_G Fsqd Refinement With I > n * Sigma(I) Only .....     Please Check
PLAT961_ALERT_5_G Dataset Contains no Negative Intensities .......     Please Check
PLAT969_ALERT_5_G The 'Henn et al.' R-Factor-gap value ...........      4.348 Note
              Predicted wR2: Based on SigI**2  0.52 or SHELX Weight  1.93
```

---

```
   0 ALERT level A = Most likely a serious problem - resolve or explain
   0 ALERT level B = A potentially serious problem, consider carefully
   4 ALERT level C = Check. Ensure it is not caused by an omission or oversight
  54 ALERT level G = General information/check it is not something unexpected

   3 ALERT type 1 CIF construction/syntax error, inconsistent or missing data
   5 ALERT type 2 Indicator that the structure model may be wrong or deficient
   6 ALERT type 3 Indicator that the structure quality may be low
  40 ALERT type 4 Improvement, methodology, query or suggestion
   4 ALERT type 5 Informative message, check
```

---

---

It is advisable to attempt to resolve as many as possible of the alerts in all categories. Often the minor alerts point to easily fixed oversights, errors and omissions in your CIF or refinement strategy, so attention to these fine details can be worthwhile. In order to resolve some of the more serious problems it may be necessary to carry out additional measurements or structure refinements. However, the purpose of your study may justify the reported deviations and the more serious of these should normally be commented upon in the discussion or experimental section of a paper or in the "special_details" fields of the CIF. checkCIF was carefully designed to identify outliers and unusual parameters, but every test has its limitations and alerts that are not important in a particular case may appear. Conversely, the absence of alerts does not guarantee there are no aspects of the results needing attention. It is up to the individual to critically assess their own results and, if necessary, seek expert advice.

### Publication of your CIF in IUCr journals

A basic structural check has been run on your CIF. These basic checks will be run on all CIFs submitted for publication in IUCr journals (*Acta Crystallographica*, *Journal of Applied Crystallography*, *Journal of Synchrotron Radiation*); however, if you intend to submit to *Acta Crystallographica Section C* or *E* or *IUCrData*, you should make sure that full publication checks are run on the final version of your CIF prior to submission.

### Publication of your CIF in other journals

Please refer to the *Notes for Authors* of the relevant journal for any special instructions relating to CIF submission.

---

**PLATON version of 11/11/2024; check.def file version of 11/11/2024**

# **Datablock FAPbBr3** - ellipsoid plot

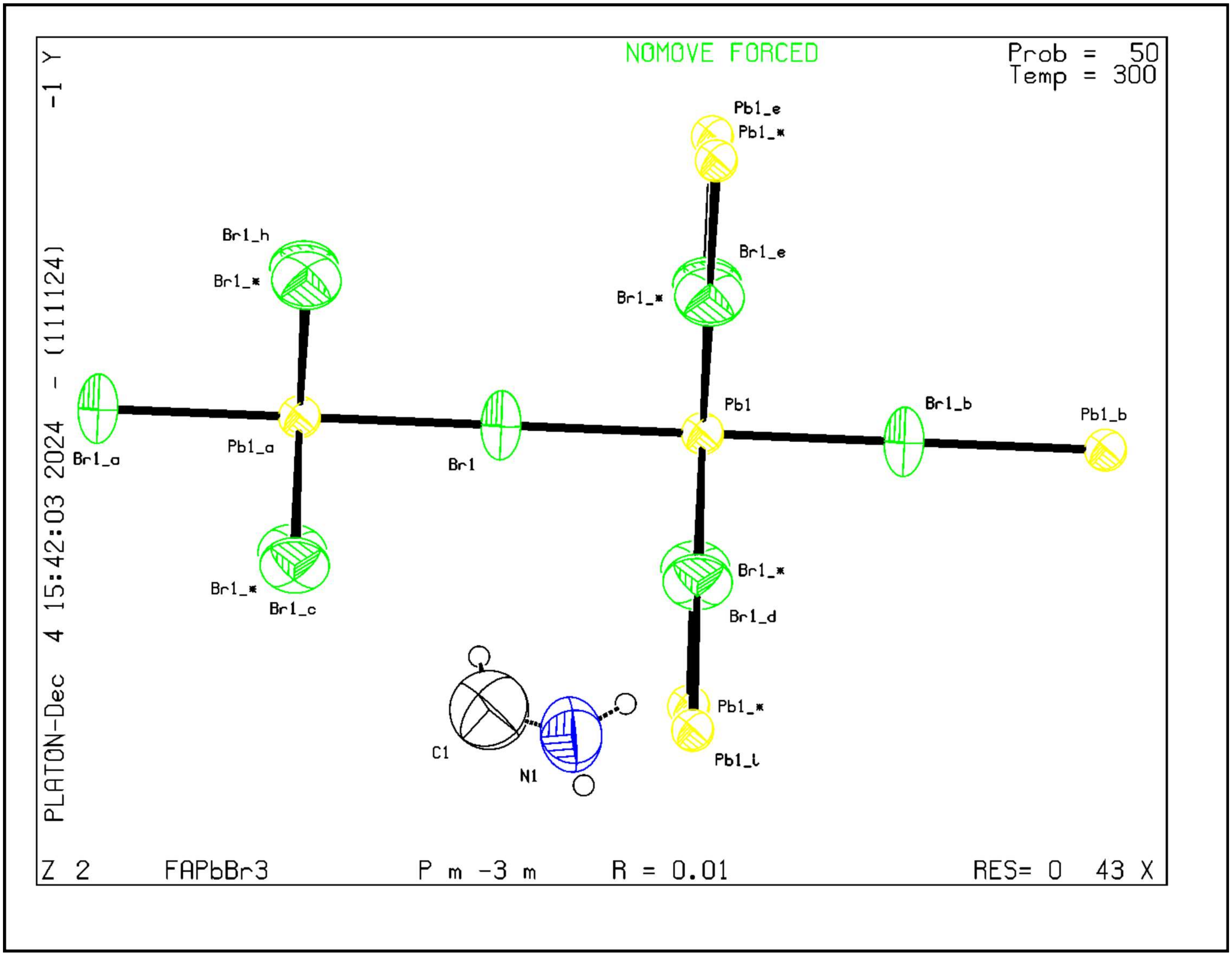


[Download CIF editor (publCIF) from the IUCr](#)
[Download CIF editor (enCIFer) from the CCDC](#)
[Test a new CIF entry](#)
