# Supplementary material for: Phonon-driven wavefunction localization enhances room-temperature single-photon purity in large hybrid lead halide perovskite quantum dots
Source: Nat Commun. 2026 Jan 23;17:1974. doi: 10.1038/s41467-026-68607-w (PMC12932643; doi:10.1038/s41467-026-68607-w)
Supplement: Supplementary file 3 — Supplementary Data 1 [file 41467_2026_68607_MOESM3_ESM.zip › 2407795_Checkcif.pdf]

No syntax errors found.
Please wait while processing ....

CIF dictionary
Interpreting this report

Datablock: CsPbBr3

Bond precision: Pb-Br = 0.0007 A Wavelength=0.71073

Cell: a=8.2575(4) b=11.7639(7) c=8.2194(4)
alpha=90 beta=90 gamma=90

Temperature: 295 K

	Calculated	Reported
Volume	798.44(7)	798.44(7)
Space group	P n m a	P n m a
Hall group	-P 2ac 2n	-P 2ac 2n
Moiety formula	Br6 Pb2, 0.667(Cs3)	Br3 Cs Pb
Sum formula	Br6 Cs2 Pb2	Br3 Cs Pb
Mr	1159.59	579.83
Dx,g cm-3	4.823	4.824
Z	2	4
Mu (mm-1)	40.519	40.519
F000	968.0	968.0
F000'	952.35	
h,k,lmax	11,16,11	11,15,10
Nref	1272	1178
Tmin,Tmax	0.087,0.263	0.636,0.825
Tmin'	0.065	

Correction method= # Reported T Limits: Tmin=0.636 Tmax=0.825
AbsCorr = GAUSSIAN

Data completeness= 0.926 Theta(max)= 30.473

R(reflections)= 0.0260(1001) wR2(reflections)= 0.0698(1178)

S = 1.077 Npar= 42

The following ALERTS were generated. Each ALERT has the format
test-name_ALERT_alert-type_alert-level.
Click on the hyperlinks for more details of the test.

Alert level C

```
PLAT029_ALERT_3_C _diffrn_measured_fraction_theta_full value Low .      0.971 Why?
PLAT041_ALERT_1_C Calc. and Reported SumFormula    Strings  Differ     Please Check
              Calc: Br6 Cs2 Pb2
              Rep.: Br3 Cs Pb
PLAT042_ALERT_1_C Calc. and Reported MoietyFormula Strings  Differ     Please Check
              Calc: Br6 Pb2, 0.667(Cs3)
              Rep.: Br3 Cs Pb
PLAT242_ALERT_2_C Low    'MainMol' Ueq as Compared to Neighbors of       Pb01 Check
PLAT906_ALERT_3_C Large K Value in the Analysis of Variance ......      3.145 Check
PLAT911_ALERT_3_C Missing FCF Refl Between Thmin & STh/L=     0.600        19 Report
                2  0  0,   4  0  0,   0  2  0,   2  9  0,   0 10  0,   4 10  0,
                6 10  0,   0 12  0,   0 14  0,   1  0  1,   0  9  1,   1  9  1,
                2  9  1,   4 10  1,   5 10  1,   6 10  1,   2  9  2,   0  8  4,
                0  8  6,
```

Alert level G

```
PLAT003_ALERT_2_G Number of Uiso or U(i,j)  Restrained non-H-Atoms           3 Report
PLAT004_ALERT_5_G Polymeric Structure Found with Maximum Dimension           3 Info
PLAT012_ALERT_1_G N.O.K.   _shelx_res_checksum Found in CIF ......     Please Check
PLAT013_ALERT_1_G N.O.K.   _shelx_hkl_checksum Found in CIF ......     Please Check
PLAT045_ALERT_1_G Calculated and Reported Z Differ by a Factor ...      0.500 Check
PLAT178_ALERT_4_G The CIF-Embedded .res File Contains SIMU Records           3 Report
PLAT188_ALERT_3_G A Non-default SIMU Restraint Value has been used     0.0100 Report
PLAT188_ALERT_3_G A Non-default SIMU Restraint Value has been used     0.0100 Report
PLAT300_ALERT_4_G Atom Site Occupancy of Cs         Constrained at     0.3333 Check
```

And 2 other PLAT300 Alerts
More ...

```
PLAT302_ALERT_4_G Anion/Solvent/Minor-Residue Disorder (Resd    2)       100% Note
PLAT720_ALERT_4_G Number of Unusual/Non-Standard Labels .........           3 Note
              Pb01    Cs02    Br03
PLAT794_ALERT_5_G Tentative Bond Valency for Pb01      (II)      .       2.75 Info
PLAT860_ALERT_3_G Number of Least-Squares Restraints ............          18 Note
PLAT870_ALERT_4_G ALERTS Related to Twinning Effects Suppressed ..          ! Info
PLAT883_ALERT_1_G Absent Datum for _atom_sites_solution_primary ..     Please Do !
PLAT912_ALERT_4_G Missing # of FCF Reflections Above STh/L=  0.600        121 Note
PLAT933_ALERT_2_G Number of HKL-OMIT Records in Embedded .res File          2 Note
                2  0  0,    4  0  0,
PLAT955_ALERT_1_G Reported (CIF) and Actual (FCF) Lmax Differ by .          1 Units
PLAT969_ALERT_5_G The 'Henn et al.' R-Factor-gap value ..........       3.243 Note
              Predicted wR2: Based on SigI**2  2.15 or SHELX Weight  6.48
```

```
 0 ALERT level A = Most likely a serious problem - resolve or explain
 0 ALERT level B = A potentially serious problem, consider carefully
 6 ALERT level C = Check. Ensure it is not caused by an omission or oversight
21 ALERT level G = General information/check it is not something unexpected

 7 ALERT type 1 CIF construction/syntax error, inconsistent or missing data
 3 ALERT type 2 Indicator that the structure model may be wrong or deficient
 6 ALERT type 3 Indicator that the structure quality may be low
 8 ALERT type 4 Improvement, methodology, query or suggestion
 3 ALERT type 5 Informative message, check
```

It is advisable to attempt to resolve as many as possible of the alerts in all categories. Often the minor alerts point to easily fixed oversights, errors and omissions in your CIF or refinement strategy, so attention to these fine details can be worthwhile. In order to resolve some of the more serious problems it may be necessary to carry out additional measurements or structure refinements. However, the purpose of your study may justify the reported deviations and the more serious of these should normally be commented upon in the discussion or experimental section of a paper or in the "special_details" fields of the CIF. checkCIF was carefully designed to identify outliers and unusual parameters, but every test has its limitations and alerts that are not important in a particular case may appear. Conversely, the absence of alerts does not guarantee there are no aspects of the results needing attention. It is up to the individual to critically assess their own results and, if necessary, seek expert advice.

Publication of your CIF in IUCr journals

A basic structural check has been run on your CIF. These basic checks will be run on all CIFs submitted for publication in IUCr journals (*Acta Crystallographica*, *Journal of Applied Crystallography*, *Journal of Synchrotron Radiation*); however, if you intend to submit to *Acta Crystallographica Section C* or *E* or *IUCrData*, you should make sure that full publication checks are run on the final version of your CIF prior to submission.

Publication of your CIF in other journals

Please refer to the *Notes for Authors* of the relevant journal for any special instructions relating to CIF submission.

PLATON version of 11/11/2024; check.def file version of 11/11/2024

Datablock CsPbBr3 - ellipsoid plot

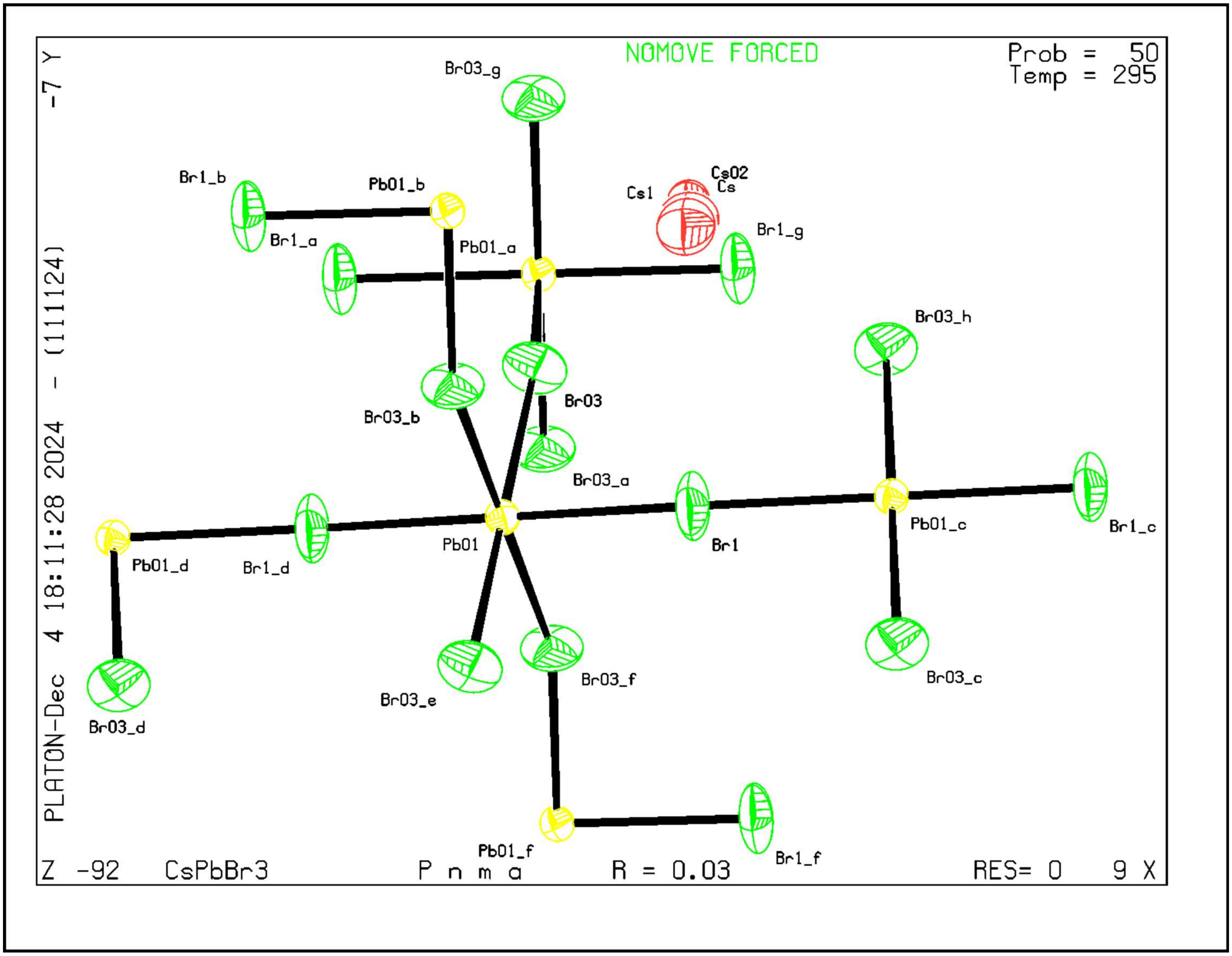

[Download CIF editor (publCIF) from the IUCr]
[Download CIF editor (enCIFer) from the CCDC]
[Test a new CIF entry]
