# Supplementary material for: Phonon-driven wavefunction localization enhances room-temperature single-photon purity in large hybrid lead halide perovskite quantum dots
Source: Nat Commun. 2026 Jan 23;17:1974. doi: 10.1038/s41467-026-68607-w (PMC12932643; doi:10.1038/s41467-026-68607-w)
Supplement: Supplementary file 3 — Supplementary Data 1 [file 41467_2026_68607_MOESM3_ESM.zip › 2407795_tables.html]

2407795


# 2407795

Table 1 Crystal data and structure refinement for 2407795.

| Identification code | 2407795 |
| Empirical formula | Br3CsPb |
| Formula weight | 579.83 |
| Temperature/K | 295.15 |
| Crystal system | orthorhombic |
| Space group | Pnma |
| a/Å | 8.2575(4) |
| b/Å | 11.7639(7) |
| c/Å | 8.2194(4) |
| α/° | 90 |
| β/° | 90 |
| γ/° | 90 |
| Volume/Å3 | 798.44(7) |
| Z | 4 |
| ρcalcg/cm3 | 4.824 |
| μ/mm‑1 | 40.519 |
| F(000) | 968.0 |
| Crystal size/mm3 | 0.064 × 0.053 × 0.033 |
| Radiation | MoKα (λ = 0.71073) |
| 2Θ range for data collection/° | 6.028 to 60.946 |
| Index ranges | -11 ≤ h ≤ 11, -15 ≤ k ≤ 15, -10 ≤ l ≤ 10 |
| Reflections collected | 12744 |
| Independent reflections | 1178 [Rint = 0.0373, Rsigma = 0.0163] |
| Data/restraints/parameters | 1178/18/42 |
| Goodness-of-fit on F2 | 1.077 |
| Final R indexes [I>=2σ (I)] | R1 = 0.0260, wR2 = 0.0675 |
| Final R indexes [all data] | R1 = 0.0306, wR2 = 0.0698 |
| Largest diff. peak/hole / e Å-3 | 1.44/-1.26 |

Table 2 Fractional Atomic Coordinates (×104) and Equivalent Isotropic Displacement Parameters (Å2×103) for 2407795. Ueq is defined as 1/3 of the trace of the orthogonalised UIJ tensor.

| Atom | *x* | *y* | *z* | U(eq) |
| --- | --- | --- | --- | --- |
| Pb01 | 0 | 5000 | 5000 | 28.47(12) |
| Cs02 | 5537(16) | 2500 | 4806(17) | 55.4(13) |
| Br03 | 2933.1(11) | 4756.2(9) | 2938.8(11) | 69.2(3) |
| Cs | 5240(30) | 2500 | 4850(20) | 74(3) |
| Br1 | -37.4(18) | 2500 | 5472(2) | 79.0(5) |
| Cs1 | 5070(30) | 2500 | 5184(18) | 77(3) |

Table 3 Anisotropic Displacement Parameters (Å2×103) for 2407795. The Anisotropic displacement factor exponent takes the form: -2π2[h2a\*2U11+2hka\*b\*U12+…].

| Atom | U11 | U22 | U33 | U23 | U13 | U12 |
| --- | --- | --- | --- | --- | --- | --- |
| Pb01 | 29.38(19) | 26.89(17) | 29.1(2) | -0.74(8) | 0.01(10) | 0.84(8) |
| Cs02 | 54(3) | 43.0(18) | 69(3) | 0 | -11(2) | 0 |
| Br03 | 56.4(5) | 94.2(6) | 56.9(5) | 5.9(4) | 30.2(4) | 5.8(5) |
| Cs | 69(6) | 85(4) | 66(5) | 0 | -1(4) | 0 |
| Br1 | 113.6(13) | 24.5(5) | 98.8(10) | 0 | -11.4(7) | 0 |
| Cs1 | 79(6) | 79(3) | 72(6) | 0 | -19(4) | 0 |

Table 4 Bond Lengths for 2407795.

| Atom | Atom | Length/Å |  | Atom | Atom | Length/Å |
| --- | --- | --- | --- | --- | --- | --- |
| Pb01 | Cs021 | 4.718(10) |  | Cs02 | Cs16 | 5.55(3) |
| Pb01 | Cs022 | 4.718(10) |  | Cs02 | Cs110 | 5.564(19) |
| Pb01 | Br033 | 2.9715(8) |  | Br03 | Cs1 | 3.683(15) |
| Pb01 | Br034 | 2.9696(8) |  | Br03 | Cs12 | 3.939(12) |
| Pb01 | Br03 | 2.9696(8) |  | Cs | Cs5 | 5.65(2) |
| Pb01 | Br035 | 2.9715(8) |  | Cs | Cs6 | 5.65(2) |
| Pb01 | Br1 | 2.9666(3) |  | Cs | Br110 | 3.856(18) |
| Pb01 | Br14 | 2.9666(3) |  | Cs | Br111 | 3.93(2) |
| Cs02 | Cs025 | 5.605(19) |  | Cs | Cs16 | 5.74(3) |
| Cs02 | Cs026 | 5.605(19) |  | Cs | Cs110 | 5.71(3) |
| Cs02 | Br036 | 4.006(11) |  | Br1 | Cs112 | 3.571(15) |
| Cs02 | Br037 | 4.006(11) |  | Br1 | Cs1 | 4.22(2) |
| Cs02 | Br038 | 3.931(7) |  | Br1 | Cs11 | 4.05(2) |
| Cs02 | Br032 | 3.931(7) |  | Cs1 | Cs110 | 5.62(2) |
| Cs02 | Br03 | 3.745(8) |  | Cs1 | Cs112 | 5.62(2) |
| Cs02 | Br039 | 3.745(8) |  |  |  |  |

1-1+X,+Y,+Z; 21-X,1-Y,1-Z; 31/2-X,1-Y,1/2+Z; 4-X,1-Y,1-Z; 5-1/2+X,+Y,1/2-Z; 61/2+X,+Y,1/2-Z; 71/2+X,1/2-Y,1/2-Z; 81-X,-1/2+Y,1-Z; 9+X,1/2-Y,+Z; 101/2+X,+Y,3/2-Z; 111+X,+Y,+Z; 12-1/2+X,+Y,3/2-Z

Table 5 Bond Angles for 2407795.

| Atom | Atom | Atom | Angle/˚ |  | Atom | Atom | Atom | Angle/˚ |
| --- | --- | --- | --- | --- | --- | --- | --- | --- |
| Cs021 | Pb01 | Cs022 | 180.0 |  | Pb0112 | Br03 | Cs023 | 83.68(17) |
| Br033 | Pb01 | Cs022 | 122.44(16) |  | Pb01 | Br03 | Cs022 | 85.1(2) |
| Br03 | Pb01 | Cs022 | 56.11(15) |  | Pb01 | Br03 | Cs023 | 88.99(17) |
| Br034 | Pb01 | Cs022 | 123.89(15) |  | Pb01 | Br03 | Cs12 | 92.2(2) |
| Br035 | Pb01 | Cs022 | 57.56(16) |  | Pb0112 | Br03 | Cs1 | 101.6(2) |
| Br03 | Pb01 | Cs021 | 123.89(15) |  | Pb01 | Br03 | Cs1 | 100.0(2) |
| Br035 | Pb01 | Cs021 | 122.44(16) |  | Pb0112 | Br03 | Cs12 | 89.9(2) |
| Br034 | Pb01 | Cs021 | 56.11(15) |  | Cs02 | Br03 | Cs023 | 92.56(19) |
| Br033 | Pb01 | Cs021 | 57.56(16) |  | Cs02 | Br03 | Cs022 | 101.78(5) |
| Br03 | Pb01 | Br035 | 90.265(7) |  | Cs022 | Br03 | Cs023 | 165.55(14) |
| Br03 | Pb01 | Br034 | 180.0 |  | Cs02 | Br03 | Cs12 | 100.4(3) |
| Br033 | Pb01 | Br035 | 180.0 |  | Cs022 | Br03 | Cs12 | 7.2(4) |
| Br03 | Pb01 | Br033 | 89.735(7) |  | Cs1 | Br03 | Cs02 | 7.6(4) |
| Br034 | Pb01 | Br035 | 89.735(7) |  | Cs1 | Br03 | Cs022 | 101.6(3) |
| Br034 | Pb01 | Br033 | 90.265(8) |  | Cs1 | Br03 | Cs023 | 92.4(3) |
| Pb016 | Cs02 | Pb017 | 77.13(19) |  | Cs12 | Br03 | Cs023 | 166.0(3) |
| Pb017 | Cs02 | Cs028 | 56.46(6) |  | Cs1 | Br03 | Cs12 | 101.17(3) |
| Pb017 | Cs02 | Cs023 | 126.8(3) |  | Cs8 | Cs | Cs19 | 88.7(4) |
| Pb016 | Cs02 | Cs023 | 126.8(3) |  | Cs3 | Cs | Cs19 | 177.3(4) |
| Pb016 | Cs02 | Cs028 | 56.46(6) |  | Br19 | Cs | Cs19 | 47.72(15) |
| Pb016 | Cs02 | Cs19 | 56.6(2) |  | Br16 | Cs | Cs19 | 38.2(4) |
| Pb016 | Cs02 | Cs18 | 59.9(2) |  | Cs19 | Cs | Cs18 | 91.7(6) |
| Pb017 | Cs02 | Cs18 | 59.9(2) |  | Pb0113 | Br1 | Pb01 | 164.92(8) |
| Pb017 | Cs02 | Cs19 | 56.6(2) |  | Pb0113 | Br1 | Cs1 | 89.61(4) |
| Cs023 | Cs02 | Cs028 | 94.9(4) |  | Pb01 | Br1 | Cs14 | 97.47(4) |
| Br03 | Cs02 | Pb016 | 91.18(10) |  | Pb01 | Br1 | Cs1 | 89.61(4) |
| Br038 | Cs02 | Pb016 | 38.76(9) |  | Pb0113 | Br1 | Cs14 | 97.47(4) |
| Br032 | Cs02 | Pb017 | 104.2(3) |  | Pb01 | Br1 | Cs114 | 97.50(4) |
| Br0310 | Cs02 | Pb016 | 154.7(4) |  | Pb01 | Br1 | Cs11 | 90.16(4) |
| Br037 | Cs02 | Pb017 | 38.84(9) |  | Pb0113 | Br1 | Cs11 | 90.16(4) |
| Br0311 | Cs02 | Pb016 | 92.6(3) |  | Pb0113 | Br1 | Cs114 | 97.50(4) |
| Br0311 | Cs02 | Pb017 | 38.76(9) |  | Pb01 | Br1 | Cs1 | 88.98(4) |
| Br038 | Cs02 | Pb017 | 92.6(3) |  | Pb0113 | Br1 | Cs1 | 88.98(4) |
| Br03 | Cs02 | Pb017 | 154.7(4) |  | Cs14 | Br1 | Cs1 | 89.8(3) |
| Br0310 | Cs02 | Pb017 | 91.18(10) |  | Cs1 | Br1 | Cs1 | 169.3(2) |
| Br037 | Cs02 | Pb016 | 104.2(3) |  | Cs114 | Br1 | Cs14 | 2.0(6) |
| Br032 | Cs02 | Pb016 | 38.84(9) |  | Cs114 | Br1 | Cs1 | 98.9(5) |
| Br038 | Cs02 | Cs028 | 41.87(16) |  | Cs114 | Br1 | Cs1 | 91.8(5) |
| Br0311 | Cs02 | Cs023 | 89.0(3) |  | Cs114 | Br1 | Cs11 | 94.8(4) |
| Br037 | Cs02 | Cs023 | 123.76(13) |  | Cs11 | Br1 | Cs1 | 173.4(4) |
| Br032 | Cs02 | Cs028 | 94.71(15) |  | Cs023 | Cs1 | Cs0214 | 95.4(4) |
| Br0310 | Cs02 | Cs023 | 45.56(12) |  | Cs0214 | Cs1 | Cs14 | 2.0(3) |
| Br0310 | Cs02 | Cs028 | 98.4(4) |  | Cs023 | Cs1 | Cs14 | 93.3(5) |
| Br037 | Cs02 | Cs028 | 94.71(15) |  | Cs0214 | Cs1 | Cs19 | 89.6(4) |
| Br03 | Cs02 | Cs023 | 45.57(12) |  | Cs0214 | Cs1 | Cs114 | 5.1(3) |
| Br038 | Cs02 | Cs023 | 89.0(3) |  | Cs023 | Cs1 | Cs19 | 175.1(3) |
| Br0311 | Cs02 | Cs028 | 41.87(16) |  | Cs023 | Cs1 | Cs114 | 90.3(3) |
| Br032 | Cs02 | Cs023 | 123.76(13) |  | Br037 | Cs1 | Cs0214 | 89.5(2) |
| Br03 | Cs02 | Cs028 | 98.4(4) |  | Br0310 | Cs1 | Cs0214 | 92.8(4) |
| Br03 | Cs02 | Br0311 | 121.5(4) |  | Br03 | Cs1 | Cs023 | 46.1(2) |
| Br0311 | Cs02 | Br038 | 83.0(3) |  | Br032 | Cs1 | Cs0214 | 89.5(2) |
| Br03 | Cs02 | Br037 | 163.7(4) |  | Br032 | Cs1 | Cs023 | 124.9(2) |
| Br037 | Cs02 | Br038 | 130.6(3) |  | Br03 | Cs1 | Cs0214 | 92.8(4) |
| Br0310 | Cs02 | Br032 | 163.7(4) |  | Br0310 | Cs1 | Cs023 | 46.1(2) |
| Br03 | Cs02 | Br032 | 78.22(5) |  | Br037 | Cs1 | Cs023 | 124.9(2) |
| Br0310 | Cs02 | Br038 | 121.5(4) |  | Br0310 | Cs1 | Br032 | 170.9(5) |
| Br032 | Cs02 | Br0311 | 130.6(3) |  | Br0310 | Cs1 | Br037 | 78.83(3) |
| Br03 | Cs02 | Br038 | 65.37(13) |  | Br0310 | Cs1 | Br03 | 92.2(5) |
| Br0310 | Cs02 | Br0311 | 65.37(13) |  | Br032 | Cs1 | Br037 | 110.0(5) |
| Br032 | Cs02 | Br038 | 64.07(10) |  | Br03 | Cs1 | Br032 | 78.83(3) |
| Br037 | Cs02 | Br0311 | 64.07(10) |  | Br03 | Cs1 | Br037 | 170.9(5) |
| Br0310 | Cs02 | Br03 | 90.3(3) |  | Br03 | Cs1 | Cs14 | 91.4(4) |
| Br032 | Cs02 | Br037 | 110.4(3) |  | Br032 | Cs1 | Cs14 | 90.7(2) |
| Br0310 | Cs02 | Br037 | 78.22(5) |  | Br0310 | Cs1 | Cs14 | 91.4(4) |
| Br0311 | Cs02 | Cs18 | 41.51(14) |  | Br037 | Cs1 | Cs14 | 90.7(2) |
| Br0311 | Cs02 | Cs19 | 94.8(3) |  | Br0310 | Cs1 | Br16 | 120.5(3) |
| Br032 | Cs02 | Cs18 | 97.6(3) |  | Br037 | Cs1 | Br16 | 63.8(3) |
| Br037 | Cs02 | Cs18 | 97.6(3) |  | Br032 | Cs1 | Br16 | 63.8(3) |
| Br0310 | Cs02 | Cs18 | 94.8(2) |  | Br03 | Cs1 | Br16 | 120.5(3) |
| Br032 | Cs02 | Cs19 | 55.58(14) |  | Br03 | Cs1 | Cs19 | 133.8(2) |
| Br0310 | Cs02 | Cs19 | 133.59(15) |  | Br037 | Cs1 | Cs19 | 55.0(3) |
| Br03 | Cs02 | Cs19 | 133.59(15) |  | Br03 | Cs1 | Cs114 | 89.3(2) |
| Br038 | Cs02 | Cs19 | 94.8(3) |  | Br037 | Cs1 | Cs114 | 92.4(3) |
| Br03 | Cs02 | Cs18 | 94.8(2) |  | Br0310 | Cs1 | Cs114 | 89.3(2) |
| Br037 | Cs02 | Cs19 | 55.58(14) |  | Br0310 | Cs1 | Cs19 | 133.8(2) |
| Br038 | Cs02 | Cs18 | 41.51(14) |  | Br032 | Cs1 | Cs19 | 55.0(3) |
| Cs18 | Cs02 | Cs023 | 89.8(3) |  | Br032 | Cs1 | Cs114 | 92.4(3) |
| Cs18 | Cs02 | Cs028 | 5.1(3) |  | Br16 | Cs1 | Br1 | 173.4(4) |
| Cs19 | Cs02 | Cs028 | 90.3(3) |  | Br19 | Cs1 | Br1 | 85.4(3) |
| Cs19 | Cs02 | Cs023 | 174.8(4) |  | Br19 | Cs1 | Cs19 | 48.7(5) |
| Cs18 | Cs02 | Cs19 | 95.4(4) |  | Br16 | Cs1 | Cs19 | 39.33(4) |
| Pb01 | Br03 | Pb0112 | 157.35(4) |  | Br1 | Cs1 | Cs19 | 134.1(4) |
| Pb0112 | Br03 | Cs02 | 94.1(2) |  | Cs114 | Cs1 | Cs14 | 3.0(3) |
| Pb01 | Br03 | Cs02 | 107.6(2) |  | Cs19 | Cs1 | Cs14 | 91.6(5) |
| Pb0112 | Br03 | Cs022 | 96.9(2) |  | Cs19 | Cs1 | Cs114 | 94.7(5) |

1-1+X,+Y,+Z; 21-X,1-Y,1-Z; 3-1/2+X,+Y,1/2-Z; 4-X,1-Y,1-Z; 51/2-X,1-Y,1/2+Z; 61+X,+Y,+Z; 71-X,-1/2+Y,1-Z; 81/2+X,+Y,1/2-Z; 91/2+X,+Y,3/2-Z; 10+X,1/2-Y,+Z; 111/2+X,1/2-Y,1/2-Z; 121/2-X,1-Y,-1/2+Z; 13-X,-1/2+Y,1-Z; 14-1/2+X,+Y,3/2-Z

Table 6 Atomic Occupancy for 2407795.

| Atom | *Occupancy* |  | Atom | *Occupancy* |  | Atom | *Occupancy* |
| --- | --- | --- | --- | --- | --- | --- | --- |
| Cs02 | 0.3333 |  | Cs | 0.3333 |  | Cs1 | 0.3333 |

Experimental

Single crystals of Br3CsPb
[2407795]
were
[.].
A suitable crystal was selected and
[]
on a
XtaLAB Synergy, Dualflex, Pilatus 300K
diffractometer. The crystal was kept at 295.15 K during data collection.
Using Olex2 [1], the structure was solved with the
Unknown
[2] structure solution program using
Unknown
and refined with the
Unknown
[3] refinement package using
Unknown
minimisation.

1. Dolomanov, O.V., Bourhis, L.J., Gildea, R.J, Howard, J.A.K. & Puschmann, H.
   (2009), J. Appl. Cryst. 42, 339-341.

Crystal structure determination of
[2407795]

**Crystal Data**
for Br3CsPb (*M*=579.83 g/mol):
orthorhombic, space group Pnma (no. 62),
*a* = 8.2575(4) Å, *b* = 11.7639(7) Å, *c* = 8.2194(4) Å,
*V*= 798.44(7) Å3,
*Z* = 4,
*T* = 295.15 K,
μ(MoKα) = 40.519 mm-1,
*Dcalc* = 4.824 g/cm3,
12744 reflections measured (6.028° ≤ 2Θ ≤ 60.946°),
1178 unique (*R*int = 0.0373, Rsigma = 0.0163) which were used in all calculations.
The final *R*1 was 0.0260
(I > 2σ(I)) and *wR*2 was 0.0698 (all data).

Refinement model description

Number of restraints - 18,
number of constraints - unknown.

Details:

```
1. Twinned data refinement
```

This report has been created with Olex2, compiled on
2024.02.16 svn.r378c4104 for OlexSys. Please
let us know
if there are any errors or if you would like to have additional features.
